# Polycyclic Aromatic Hydrocarbon-Degrading Bacteria in Three Different Functional Zones of the Cities of Moscow and Murmansk

**DOI:** 10.3390/microorganisms10101979

**Published:** 2022-10-06

**Authors:** Olesya I. Sazonova, Olga Gavrichkova, Anastasia A. Ivanova, Kirill V. Petrikov, Rostislav A. Streletskii, Dmitriy A. Sarzhanov, Maria V. Korneykova, Andrey I. Novikov, Viacheslav I. Vasenev, Kristina V. Ivashchenko, Marina V. Slukovskaya, Anna A. Vetrova

**Affiliations:** 1 Federal Research Center “Pushchino Scientific Center for Biological Research of the Russian Academy of Sciences”, 142290 Pushchino, Russia; 2 Research Institute on Terrestrial Ecosystems, National Research Council, 05010 Porano, Italy; 3 Faculty of Soil Science, Laboratory of Ecological Soil Science, Lomonosov Moscow State University, 119991 Moscow, Russia; 4 Agrarian and Technological Institute, Peoples’ Friendship University of Russia (RUDN University), 117198 Moscow, Russia; 5 Institute of North Industrial Ecology Problems Subdivision of the Federal Research Center “Kola Science Centre of Russian Academy of Science”, 184209 Apatity, Russia; 6 I.V. Tananaev Institute of Chemistry and Technology of Rare Elements and Mineral Raw Materials, Kola Science Centre, Russian Academy of Sciences, 184209 Apatity, Russia; 7 Soil Geography and Landscape Group, Wageningen University, 6707 Wageningen, The Netherlands; 8 Laboratory of Nature-Inspired Technologies and Environmental Safety of the Arctic Region, Kola Science Centre, Russian Academy of Sciences, 184209 Apatity, Russia

**Keywords:** PAH-degrading bacteria, polycyclic aromatic hydrocarbons, urban ecosystems, functional zones, biotopes, dust, microbiomes, adaptation

## Abstract

We performed a comparative study of the total bacterial communities and communities of cultivable polycyclic aromatic hydrocarbons (PAH)-degrading bacteria in different functional zones of Moscow and Murmansk that were formed under the influence of the PAH composition in road and leaf dust. The PAHs were determined by high-performance liquid chromatography (HPLC); the bacterial communities’ diversity was assessed by metabarcoding. The degraders were isolated by their direct plating on a medium with the PAHs. The PAH total quantity declined in the leaf dust from the traffic to the recreational zone. For the road dust, a negative gradient with pollution was observed for *Rhodococcus* and *Acinetobacter* degraders and for their relative abundance in the microbiome for the functional zones of Moscow. The opposite effect was observed in the Murmansk leaf dust for the *Rothia* and *Pseudomonas* degraders and in the Moscow road dust for *Microbacterium.* The PCA and linear regression analyses showed that the *Micrococcus* degraders in the dust were sensitive to anthropogenic pollution, so they can be used as a tool for monitoring anthropogenic changes in the biosphere. The data on the degraders’ and microbial communities’ diversity suggest that minor degrading strains can play a key role in PAH degradation.

## 1. Introduction

The pollution of the urban ecosystem with polycyclic aromatic hydrocarbons (PAH) is a significant problem. Their accumulation in the environment poses a serious hazard since many of these compounds are toxic and carcinogenic [1,2,3]. PAHs are part of fine dust, which is one of the most harmful pollutants that is associated with the urban environment. It is known that fine dust can serve as a carrier of PAHs via the movement of air masses. Airborne PAHs can be deposited on the surface of various biotopes (paved road surfaces, concrete coverings, the surface of building walls, leaf surfaces, soil, etc.), and they can also be carried by the wind to other areas [4]. The pollution of ecosystems with PAHs has been studied mainly on one biotope, e.g., soil, phylloplane, or air [5,6,7,8]. Urban soils and plants, on the one hand, adsorb and accumulate the air pollution; on the other hand, due to their physiological functions, they contribute to its cleansing. The phyllosphere is the largest link in the biosphere–atmosphere interface interaction on earth [9]. The phylloplane or the leaf’s surface is known for its excellent capacity to accumulate pollutants, thereby making it a good indicator of the environmental quality. It is known that the amount of PAHs that are accumulated on the phylloplane correlates with that which is contained in the atmospheric air [10,11,12]. The main factor influencing the concentration of the PAHs on the plant tissues is considered to be the plant’s species, which is determined probably by the unique morphological and chemical features of the leaves at the species level [13,14,15]. The microbial community that is hosted by phylloplane, its activity, and its taxonomic structure was demonstrated to be sensitive to the distance from it to the pollution source in *Betula pendula* Roth, thereby making it a good indicator of the anthropogenic load [16]. In this study, we further explore the sensitivity of Betula’s phylloplane, extending the research to different climatic conditions, and taking into account the different functional zones of the cities.

The distribution of the PAHs that are in cities is influenced by multiple factors such as the emission sources, topography, and meteorological conditions. The layout structure of modern cities is complex and diverse, but there are several main functional zones: central, industrial, recreational, residential, external-transport, communal, and warehouse [17]. As a rule, the functional zoning determines the degree of the anthropogenic impact on the ecosystems and can strongly influence the structure and functioning of their biological components, vegetation, and microorganisms [18,19].

Apart from the PAHs, fine dust may include microbial communities, which are a key part of most ecosystems. The concentration and the type of pollutant influence the number and taxonomic representation of the degrading bacteria in the microbial communities [20,21]. The presence of the PAHs, due to their toxic effects, can inhibit the development of the microbiota, thereby reducing the diversity of the microbial communities [22]. In turn, the PAH-degrading bacteria that are present in the community ensure that there is a decrease in the concentration of these compounds and, consequently, they perform the cleansing function in the environment. Previous research on the PAH-degrading bacteria in the phylloplane has demonstrated the presence of the following bacteria: *Alcaligenes*, *Bacillus*, *Serratia* [23], *Sphingomonas*, *Pseudomonas* [24], *Microbacterium*, *Rhizobium*, *Deinococcus* spp. [5,13,25], and *Mycobacterium* [14,15]. The role of these bacteria in the degradation of the PAHs in soils has been extensively studied [26,27]. Instead, studies of the bacteria degrading the PAHs and inhabiting the dust of the urban sealed surfaces are still missing.

Similar to the PAH ecosystems’ pollution, the diversity of the degrading bacteria has been studied on a single biotope; however, no comparative studies have been conducted [28,29,30]. According to [24], when using leaves as a bioindicator of the PAH pollution levels in the urban air, it is recommended to characterize the bacterial communities of the phyllosphere, especially by the number and types of bacteria that decompose the PAHs.

Depending on the level and composition of the pollution in certain functional urban areas, the microbial communities of the PAH-degrading strains in various biotopes will have specific compositions and functional activities. The investigation of the microbial communities and the search for the relationship of their composition with the PAH content are important scientific problems. In addition, the PAH concentrations in the environment have been found to vary between cities, with there being relatively high levels of them in northern cities and low concentrations of them in the south [6,31,32]. The climate–pollution interaction is influencing the microbial communities and this remains to be, still, largely overlooked. For this reason, we decided to conduct a study on the biotopes of the functional zones of two cities, Murmansk and Moscow, which are located in different climatic zones.

The aim of this work was to perform a comparative study of the cultivable PAH-degrading bacterial communities in two biotopes (road dust and leaf dust) which are formed under the influence of the qualitative and quantitative composition of three functional urban zones (traffic, residential, and recreational) of two cities that differ in their climate. A combination of different approaches were performed; a study of the generic diversity of the PAH-degrading bacteria and the taxonomic profiling of these genera in the microbial community with the qualitative and quantitative composition of the PAHs revealed the links between the biological and chemical characteristics in the studied biotopes. The use of the classical method of microbiology in combination with the taxonomic profiling of the microbial community and the chemical composition of the PAHs in the different biotopes gave the possibility to judge the functional capabilities of the microbial communities.

## 2. Materials and Methods 

### 2.1. Climatic Characteristics of Research Areas and Site Description 

The study was carried out in two big industrial cities of the Russian Federation, Murmansk and Moscow, which are located, respectively, in two climatic zones: the subarctic and the temperate continental. Murmansk is the biggest city in the world beyond the Arctic Circle. The city is located on the shore of the ice-free Kola Bay, 50 km from the exit to the Barents Sea. The climate of Murmansk is formed by its proximity to the Barents Sea, the influence of which is enhanced by the warm North Atlantic current and, according to its climatic conditions, Murmansk is equated to the territories of the Far North. In summer, Murmansk has a polar day that lasts 62 days. The average annual temperature is −0.4 °C. The average annual wind speed is 4.4 m/s. On average, about 601 mm of precipitation falls per year [33,34]. Moscow is the largest city in Russia. It stands on the Moskva River in Central Russia. Moscow’s climate is temperate continental, which is transitional from the mild European climate to the sharply continental Asian climate. The continentality increases from the northwest to the southeast. Moscow features a clear seasonality of moderately cold winters and warm summers. The climatic conditions of the city are influenced by the Gulf Stream with cyclones that come from the Mediterranean and the Atlantic. The average annual temperature is +5.8 °C, the average annual wind speed is 2.3 m/s, and the average annual air humidity is 76%. During the year, 600–800 mm of atmospheric precipitation falls in Moscow and the adjacent territory, with most of it falling in the summer months [33,34]. The sampling was carried out in the summer of 2021. To ensure that there was similar seasonality and a similar phenological stage of the vegetation, the sampling in Moscow was conducted earlier than it was conducted in Murmansk. The comparative analysis of the meteorological conditions between the two cities is illustrated in Figure S1, and it describes the two-week period before the sampling was conducted. The average air temperature (*T*_av_) in Moscow and the average maximum (*T*_max_) and minimum (*T*_min_) air temperatures were two degrees lower than they were in Murmansk (15 °C, 21 °C, and 10 °C, respectively). Moscow was characterized by higher precipitations.

The functional zones of the cities that differed in the degree of the anthropogenic load were chosen for sampling: a zone of intensive traffic (traffic zone), a residential area with limited traffic (residential zone), and an urban park area away from the main highways (recreational zone). The geographic coordinates of the selected sites are as follows: the traffic zone, 68.960117 N, 33.064084 E (Murmansk) and 55.738328 N, 37.620061 E (Moscow); the residential zone, 68.978944 N, 33.093556 E (Murmansk) and 55.651983 N, 37.499363 E (Moscow); the recreational zone, 68.941098 N, 33.119497 E (Murmansk) and 55.833000 N, 37.549794 E (Moscow) (Figure S2). Sites were chosen according to the traffic load data and a visual assessment of the area. The anthropogenic gradient was confirmed by analyses which were conducted on the chemical composition of the dust that was collected from the leaves at each site [35].

### 2.2. Sampling

The object of our research was dust. In each of the three functional zones, the dust samples were taken from the surfaces of two biotopes: the leaf surfaces and the paved road surfaces.

The road dust sampling was carried out by sweeping the material with a sterile brush from a 1 m^2^ area. To obtain the samples with dust particles that were less than 100 µm (further in the text “road dust”), the collected material was sieved through a sterile sieve. The samples that were obtained were weighed. The road dust was collected from 3 representative plots of 1 m^2^ in each functional zone near the selected birches. The samples were taken in three independent repeats.

The leaf dust samples were taken from the surface of leaves of *Betula pubescens* Ehrh. (in the text, this is referred to as “leaf dust”) in accordance with the procedure that is described in [16]. In each functional zone, the birch leaves were sampled from 3 mature trees of the same age and physiological state. The sampling considered the entire circumference of the crown at a height 1.5–2.5 m. In total, 150 leaves were collected from each birch. The samples were taken in three independent repeats.

### 2.3. Chemical Analysis

#### 2.3.1. Sample Preparation of Road Dust

The PAHs were initially extracted from 2 g of road dust using 50 mL of methylene chloride and separated by high performance liquid chromatography (HPLC) using an Agilent 1260 system (USA) with the fluorescence detector. We analyzed 13 PAH types of the 16 that are presented in the Priority Pollutants List [36]: fluorene, acenaphthene, phenanthrene, anthracene, fluoranthene, pyrene, benz[a]anthracene, chrysene, benzo[b]fluoranthene, benzo[k]fluoranthene, benzo[a]pyrene, dibenz[a,h]anthracene, and benzo[g,h,i]perylene. The detection limit for the PAHs in these samples which was acquired using the combination of PAH extraction and HPLC detection methods was 0.05 µg/kg. The recovery rates varied between 90 and 97%. The PAH Calibration Mix (Merck, Germany) was used as a standard. The components were quantified by an absolute calibration curve method. 

#### 2.3.2. Sample Preparation of Leaf Dust

The samples of the dust from the surface of the birch leaves were obtained by us carefully washing the leaves. The leaves were placed in flasks containing 30 mL of deionized water which were put on an orbital shaker for 10 min. The obtained solution was filtered through a 100-µm mesh to remove the large impurities. The PAHs of 10 mL of methylene chloride were extracted from the filtered solution (about 30 mL). Each sample was prepared in triplicate. The leaves were scanned, and their area was determined. The resulting samples were analyzed using the HPLC method that is described in Section 2.3.1.

### 2.4. Microbiome Analysis of Dust

The DNA extractions from the dust that was collected from the paved road surface and the dust that was deposited on the leaf surface were carried out using the DNeasyPowerSoil Pro Kit and the DNeasyPowerWater Kit (QIAGEN, Germany), respectively, in accordance with the manufacturer’s protocol as described in [37]. The polymerase chain reaction (PCR) amplification, the library preparations for the next-generation sequencing, and the Illumina MiSeq sequencing of the bacterial 16S rRNA genes were conducted by Sequentia Biotech SL (Barcelona, Spain). The sample preparation of the leaf dust was performed as described in [37]. The bioinformatics analysis was performed as described in [16]. The raw read sequences were quality-trimmed while the adaptor sequences were removed using Trimmomatic v0.32360. The sequence quality procedure was performed using the FastQC toolkit (Babraham Bioinformatics, Cambridge, UK). A quality check was performed on the raw sequencing data, removing the low-quality bases and adapters, while preserving the longest high-quality part of the reads. The minimum length that was established was 50 bp and the quality score was 20, which increases the quality and reliability of the analysis. For the taxonomic profiling and quantification of the samples, the proprietary software GAIA (version 2.02, Sequentia Biotech, Spain) was used. GAIA works as follows: (1) each pair of reads is aligned against one or more reference databases, and the best alignments are extracted; (2) a Lowest Common Ancestor (LCA) algorithm is applied to the best alignments; (3) the identity and coverage thresholds are applied to the alignments; (4) the taxonomy is summarized and reported. The database that was used for this analysis included the 16S sequences that were obtained from the NCBI “nr” database. All of the sequences from each sample were clustered into OTUs based on their sequence similarity (97% identity).

### 2.5. Isolation and Identification of Hydrocarbon-Oxidizing Bacteria

#### 2.5.1. Sample Preparation 

(1) Road dust. An amount of 500 mg of mixed dust from a paved road surface of each repeat from one functional urban area was introduced into a test tube containing 4.5 mL of a sterile saline solution (0.9%). The resulting suspension was used for further microbiological analyses.

(2) Leaf dust. Leaves with an area of about 1000 cm^2^ of each repeat from one functional urban area were placed in a flask containing 50 mL of saline solution (0.9%) and intensively mixed on an orbital shaker for 10 min. The resulting suspension was used for further microbiological analyses.

The total count of the cultured bacteria (CFU/g of dust) for each sample was determined by the direct counting of the colonies that were grown on a Luria-Bertani (LB) agar [38] at 28 °C after 5 days. The experiments were carried out in triplicate. 

#### 2.5.2. Isolation of Hydrocarbon-Oxidizing Bacteria

For the isolation of the hydrocarbon-oxidizing bacterial strains, a direct plating was carried out from the suspensions that were obtained in Section 2.5.1 on the Evans mineral medium [39] with the addition of hydrocarbons as a sole source of carbon and energy. Naphthalene, phenanthrene, and anthracene were added to the lid of an inverted Petri dish. Benzo[a]pyrene was added by rubbing it on the surface of the agar (final concentration, 0.5 mg/mL). The morphologically distinguishable colonies of cells were inoculated to separate the colonies to obtain the pure cultures. The isolated pure strains were repeatedly tested for the hydrocarbon-oxidizing activity by growing them in Evans mineral medium with one of the four above-mentioned polycyclic aromatic substrates at 24 °C for 10 days. Salicylate was also used to test the substrate specificity of the isolated degrading strains since it is a key intermediate of the degradation pathway for many PAHs. Salicylate was added to the pre-cooled Evans mineral medium to a final concentration of 1 g/L. The reliability of the results was confirmed by the growth of the studied strains in a liquid mineral medium with the corresponding PAHs and the absence of growth on mineral agar without PAHs as a carbon source.

#### 2.5.3. DNA Manipulations

The bacterial genomic DNA was isolated using the GeneJET Genomic DNA Purifcation Kit (Thermo Scientific, Waltham, MA, USA) according to the manufacturer’s protocol. The purity and concentration of the DNA that was obtained were determined through 260/280 nm absorbance measurements using a NanoDrop spectrophotometer 2000 (Thermo Scientific, USA) according to the manufacturer’s protocol. The DNA electrophoresis was performed in 1–2.5% agarose in 0.5× Tris-borate–EDTA buffer according to the standard technique [35]. The visualization of the DNA was carried out by adding ethidium bromide to the agarose at a final concentration of 0.5 μg/mL. The polymerase chain reaction was performed using a GeneAmp PCR System 9700 cycler (Applied Biosystems, Waltham, MA, USA). The reaction was carried out under standard conditions at a final concentration of each dNTP of 200 μM and 2 mM MgCl_2_ (Sigma, St. Louis, MI, USA). The genomic fingerprinting (REP-PCR) was carried out with 5’-GTGGTGGTGGTG GTG-3’ primer according to [40]. The reaction mixes (25 µL) that were used for (GTG)_5_-PCR were prepared using 100 ng template DNA, 2 µM concentration of primers, 1 × PCR buffer, 200 µM of each dNTP, 3 mM MgCl_2_, 5% dimethyl sulfoxide, 0.1 mg/mL bovine serum albumin, and 1.25 U Taq DNA polymerase (Thermo Scientific, USA). After the gel electrophoresis, we used the PyElph software [41] to analyze the gel images of the genetic DNA markers and generated the phylogenetic trees based on the information that was available in the gel image using the Neighbor Joining method. 

#### 2.5.4. Taxonomic Identification of Hydrocarbon-Oxidizing Strains

The identification of the isolated hydrocarbon-oxidizing bacteria was carried out via the determination of the primary nucleotide sequences in a fragment of 16S rRNA gene with 27f (5’-AGAGTTTGATCMTGGCTCAG-3’) and 1492r (5’-TACGGYTACCTTGTTACGACTT-3’) primers [42]. The nucleotide sequence of the amplicons was determined using an Applied Biosystems 3130 × 1 sequenator using the sequencing kit BigDye v.3.1 (Thermo Scientific, Waltham, MA, USA). The identities of the nucleotide sequences were analyzed using the BLASTN program [43].

### 2.6. Statistics

The statistical analysis and visualization of the experimental data were carried out using the Microsoft Office Excel software. Descriptive statistics were used to determine the mean and standard error. The significant differences in the variables between the studied sites were examined by a one-factor analysis of variance (ANOVA). The hierarchical clustering was performed in https://software.broadinstitute.org/morpheus (accessed on 14 July 2022) using the Pearson correlation. The hierarchical clustering recursively merged the objects based on their pair-wise distance. A heat map was constructed to explore the correlations between the detected hydrocarbon-oxidizing bacteria at the genus level (relative abundance) and sites/biotopes. Another heat map was constructed to explore the correlations between the chemical parameters (relative abundance of PAH concentrations) and sites/biotopes. The principal component analysis (PCA) was performed in XLSTAT using R-package 4.2.1 (R Development Core Team 2022, Vienna, Austria). The matrix of the paired correlation was used to search for the main components based on the connectivity of the properties of the two cities functional zones (PAH concentration and degraders) with each other using the Pearson correlation coefficient. A linear regression analysis was performed to assess a possible correlation between the normalized PAH concentration and the quantitative indicators of the degraders.

## 3. Results and Discussion

There is a growing interest on the spatial distribution of PAHs depending on the functional zones of the city in which they are located [25,44]. In the present work, we used an integrated approach to study the biodiversity of the degraders and the PAH pollution in the dust of two biotopes (leaves of the woody plant *Betula pubescens* Ehrh. and paved road surfaces) in three functional zones of two cities that are located in a temperate continental and subarctic climates.

### 3.1. Characteristics of Collected Samples from Two Biotopes of Murmansk and Moscow Functional Zones

The analysis of the areal density of the collected dust showed the presence of a gradient only for the dust from the leaf surfaces of both of the cities (Figure S3). However, the directions of the gradients were opposite. It should be noted that in Moscow, in the traffic zone, the areal density of the road dust was higher than that of the leaf dust. In Murmansk, an opposite effect was observed. It is known that leaves are one of the indicators of the air quality [20]. Given the standard deviations, the gradient in the amount of leaf dust in Moscow is not significant, unlike in Murmansk, where in the traffic zone, the total density of the collected dust was higher than this by more than twofold. This is due to the fact that Murmansk is one of the few large cities in Russia where thermal power plants and boiler stations using fuel oil are still in operation. When the fuel oil is burnt, sulfur and carbon oxides, nitrogen dioxide, benz[a]pyrene, fuel oil ash, and products of mechanical underburning of the fuel are released into the atmosphere together with flue gases [45]. It is probable that the combination of pollution factors caused the total number of cultivated bacteria in the dust of the investigated biotopes of the Murmansk functional zones to be lower than they are in Moscow (Figure S4). At the same time, the concentration of the bacteria in the leaf dust in the traffic zone of both of the cities was higher when it was compared to that of the other functional zones. The main pollutants of the traffic zones are exhaust gases, whose components can probably be a substrate for the growth and development of the hydrocarbon-oxidizing bacteria of the microbial communities in various ecosystem biotopes. This is confirmed by the number of PAH-degrading bacteria that were observed in the traffic zones of both of the cities, which was on average 4–6% of the total pool of the cultivated bacteria, exceeding the that of the other zones by more than two times.

### 3.2. Isolation and Characterization of PAH-Degrading Bacteria

When characterizing a bacterial community, researchers often use enrichment cultivation to isolate the strains that have a certain specificity with respect to a single factor, e.g., bacteria degrading hydrocarbons [30]. In our opinion, it is not the most active degraders that survive and accumulate, but those which adapt best to certain environmental conditions. It may well be that the oxidative activity of some minor groups is high, but some other factors prevent them from becoming the dominant taxon in the population under the enrichment culture conditions [27]. Long-term cultivation can be associated with co-metabolism when the members of the consortium utilize the products of the microbial lysis. Since the bacteria in the consortium are interdependent, some strains that participate in the utilization of the pollutant and predominate in the community of enrichment cultures may be incapable of growing on solid selective media as individual colonies. The latter fact can be explained not only by the occurrence of cooperative pollutant degradation by different strains, but also by syntrophy involving different metabolic reactions [30]. In the case of direct plating, it is the active community degraders that are isolated. For this reason, in the present work, the PAH-degrading bacteria were isolated by direct plating on a mineral agarized medium containing one of the following PAHs as a source of carbon and energy: naphthalene, phenanthrene, anthracene, or benzo[a]pyrene. Fossil fuel combustion has been shown to be a major source of two- and three-ring PAHs in urban ecosystems [5]. For example, in Beijing, the roadside soils were dominated by 2–3-ring PAHs, which passed from the soil to the air gas phase which is a process that was favored by the high summer temperatures [5]. Benzo[a]pyrene is one of the most toxic PAHs, it has the strongest carcinogenic activity and, by the degree of its impact on the body, is attributed to the hazard class I. It is also known that mean benzo[a]pyrene concentrations exceed the maximum permissible concentrations in the Russian Federation by 3–5 times on average [6]. An analysis of the literature, including the above works, has determined the range of the PAHs that were chosen for the present study.

In the course of the work, 95 hydrocarbon-oxidizing bacteria were isolated from the Murmansk dust samples (34 from the paved road surfaces and 61 from the leaf surfaces) and 88 bacteria from the Moscow dust samples (65 from the paved road surfaces and 23 from the leaf surfaces). It should be noted that the lowest number of the degrading strains (9.3%) were isolated on an agarized medium with anthracene as the sole source of carbon and energy. The highest number of degraders were from the media with benzo[a]pyrene or phenanthrene, which represented 39.3% and 32.3% concentrations, respectively. The degrading bacteria that was grown on the individual substrates were additionally tested for the degradation of the other PAHs that were used in the work. In addition, salicylate, a key metabolite for the degradation of naphthalene, phenanthrene, and anthracene [46], was used as a carbon and energy source. Most of the strains were capable of degrading 3–5 of the substrates that were used (Table 1).

A cross-check of the substrate specificity revealed that the majority of the strains that were isolated in this work were capable of oxidizing one of the most toxic PAHs, benz[a]pyrene (95.7%). This effect demonstrates the adaptive capacity of the microbial community to the key pollutants of the urban ecosystem.

### 3.3. Taxonomic Diversity of Hydrocarbon-Oxidizing Bacteria of Murmansk and Moscow Functional Zones

The isolated PAH-degrading bacteria were identified by determining the primary nucleotide sequence of the 16S rRNA gene fragment. The strains belonged to the following genera: *Rhodococcus*, *Bacillus*, *Micrococcus*, *Methylorubrum*, *Acinetobacter*, *Arthrobacter*, *Pseudomonas*, *Microbacterium*, *Paenibacillus*, *Rothia*, *Pantoea*, *Phyllobacterium*, *Pseudarthrobacter*, *Stenotrophomonas*, *Nocardioides*, *Exiguobacterium*, *Methylobacterium*, *Brevundimonas*, *Staphylococcus*, *Streptomyces*, *Leifsonia*, *Dermacoccus*, *Gordonia*, *Rhizobium*, *Cellulomonas*, *Curtobacterium*, *Kocuria*, *Deinococcus*, *Rahnella*, *Enterobacter*, *Moraxella*, and *Shinella*. In the Murmansk dust samples, the diversity of the genera to which the isolated PAH degraders belong was two times higher than when it was compared to the samples from Moscow (Table 1). Bacteria of the genus *Micrococcus* were detected in the leaf dust samples in all of the functional zones of both of the cities (Figure S5I). The Vienna S5 diagram (Figure S5II) demonstrates the presence of the common genera for the residential (*Pseudomonas*) and recreational (*Bacillus*, *Micrococcus*) zones in the different Murmansk and Moscow biotopes. No common genera of degrading bacteria were identified for the traffic zone dust between the cities. We carried out a parallel analysis of the taxonomic diversity of the microbial communities from the two biotopes (road dust and leaf dust) of the three functional zones (residential, recreation, and traffic) in Moscow and Murmansk. The range of the total relative abundance of the PAH-degrading genera varied from 2.72% to 10.89% for the dust samples from Murmansk and from 2.51% to 7.12% for the dust samples from Moscow. The relative abundance of each of these genera in the microbiomes of the dust samples from Moscow ranged from 0.01 to 4.00%; for samples from Murmansk, this ranged from 0.01% to 4.20% (Table S1). It should be noted that in the microbial community of the Murmansk road dust in the anthropogenic gradient from the traffic zone to the recreational zone, we detected a decrease in the total representation of the genera to which the detected strains of the PAH degraders belong.

To establish the phylogenetic relationship between the strains that were isolated from the road and leaf dust and to study their diversity, we performed the REP-PCR with the primer (GTG)_5_ [40]. The phylogenetic trees that were generated by PyElph using the Neighbor Joining method are presented in Figure S6 As a result, we identified two large clades among the hydrocarbon-oxidizing strains from both the Moscow and Murmansk dust samples (Figure S6). Each clade, in turn, was subdivided into several subclades; in the case of Murmansk strains, this division was more pronounced. It should be noted that we found closely related strains among the bacteria that were isolated from the dust of two functional zones of Moscow (recreational and residential zones)—M GP44PH and M RP27PH1 of the genus *Paenibacillus*. Despite the fact that the functional areas in both of the cities are at a distance from one another, a more intensive Moscow traffic, a different wind rose, and a larger population apparently promoted the movement of bacteria between the sites. Additionally, in Moscow, we identified two groups of closely related strains that were isolated from the two biotopes: in the traffic zone, these were M TP75aPH and M TL38N2 (*Bacillus*); in the recreational zone, these were M GP8BP and M GL1PH (*Micrococcus*). This indicates the movement of bacteria between the biotopes within the same functional zone. No such effect was detected for the dust samples that were collected in Murmansk.

### 3.4. Hydrocarbon-Oxidizing Bacteria of Leaf Dust of the Cities’ Functional Zones 

**Figure 1:**
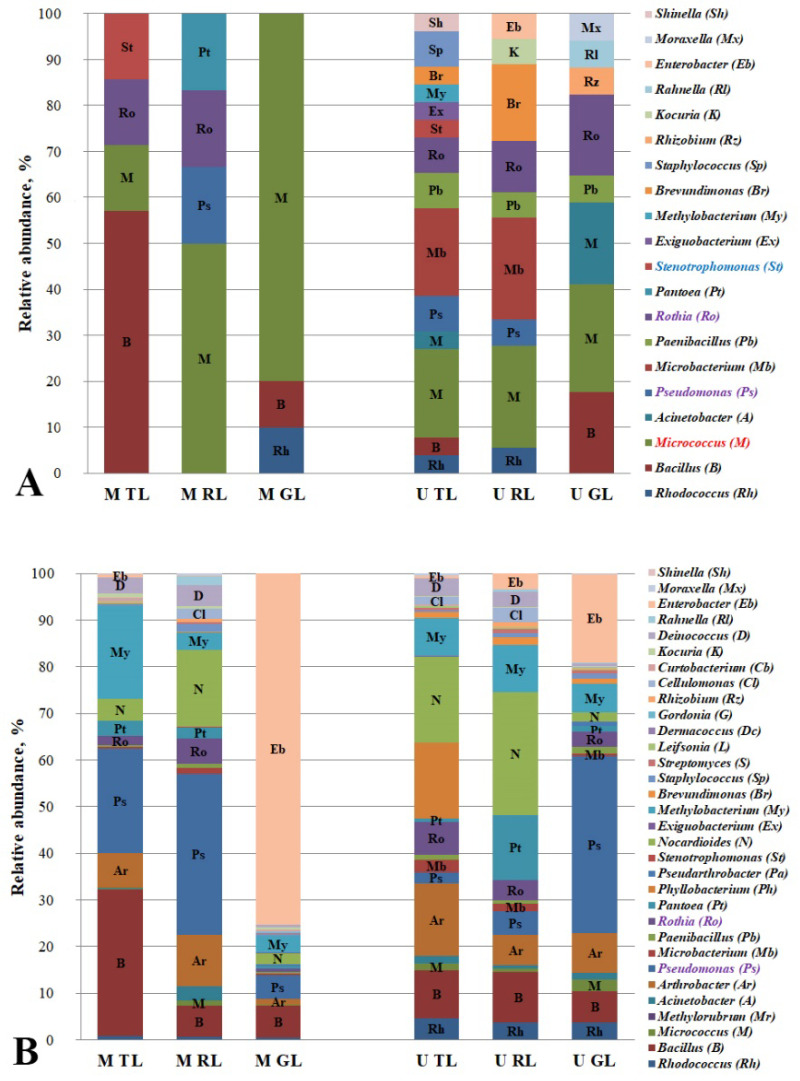
Relative abundance of isolated hydrocarbon-oxidizing bacteria (**A**) and these genera in microbiome (**B**) of leaf dust in Murmansk and Moscow functional zones. M TL, leaf dust from Moscow traffic zone; M RL, leaf dust from Moscow residential zone; M GL, leaf dust from Moscow recreational zone; U TL, leaf dust from Murmansk traffic zone; U RL, leaf dust from Murmansk residential zone; U GL, leaf dust from Murmansk recreational zone.

Phyllosphere bacteria can be active degraders of the gaseous and deposited PAHs that are on leaves [23,47,48]. The most effective degraders of phenanthrene in the phyllosphere of *Ixora* spp. growing on the roadsides in Thailand were the bacteria of the genera *Pseudomonas*, *Microbacterium*, *Rhizobium,* and *Deinococcus* [48]. The PAH-degrading bacteria of the genera *Acinetobacter*, *Pseudomonas*, *Pseudoxanthomonas,* and *Mycobacterium* which were isolated from the ornamental plant phylloplane in Bangkok accounted for 1–10% of the microbial community. These bacteria possess a high ability to degrade PAHs, including low-molecular-weight (acenaphthene, acenaphthylene and anthracene) and high-molecular-weight (fluoranthene, pyrene and benzo[a]pyrene) compounds [24]. An analysis of the relative representation of the identified genera of the PAH-degrading bacteria in the dust from the leaves of the Moscow and Murmansk functional zones is shown in Figure 1.

The isolated strains of the PAH degraders from the leaf dust belonged to the following genera: Rhodococcus, Bacillus, Micrococcus, Acinetobacter, Pseudomonas, Microbacterium, Paenibacillus, Rothia, Pantoea, Stenotrophomonas, Exiguobacterium, Methylobacterium, Brevundimonas, Staphylococcus, Rhizobium, Kocuria, Rahnella, Enterobacter, Moraxella, and Shinella. Figure 1A shows a greater variety of the genera of the isolated PAH degraders in the dust from Murmansk leaf surfaces. In the anthropogenic gradient from the traffic zone to the recreational zone of Murmansk, we observed an increase in the relative abundance of the degrading bacteria belonging to the genera Micrococcus (highlighted in red in Figure 1A) and Rothia and a decline in those belonging to Pseudomonas. In the leaf dust samples from Moscow, a positive trend was also observed with respect to the PAH-degrading bacteria of the genus Micrococcus. The genera Micrococcus and Rothia microbial PAH degraders are probably sensitive to the pollution of the traffic zone, in contrast to Pseudomonas strains which demonstrated a resistance to this. From Figure 1B, a clear opposite effect can be seen for the Rothia and Pseudomonas that were isolated from the leaf dust in Murmansk (highlighted in lilac in Figure 1A,B). The presence of the Stenotrophomonas degraders in the phylloplane of the traffic zones only can be an indicator of the PAH contamination level. In a microbial community, each genus consists of the representatives bearing different functional characteristics, so the results of the microbiome analysis for the above genera differ from the data of the direct plating.

### 3.5. Hydrocarbon-Oxidizing Bacteria of Road Dust of the Cities’ Functional Zones 

**Figure 2:**
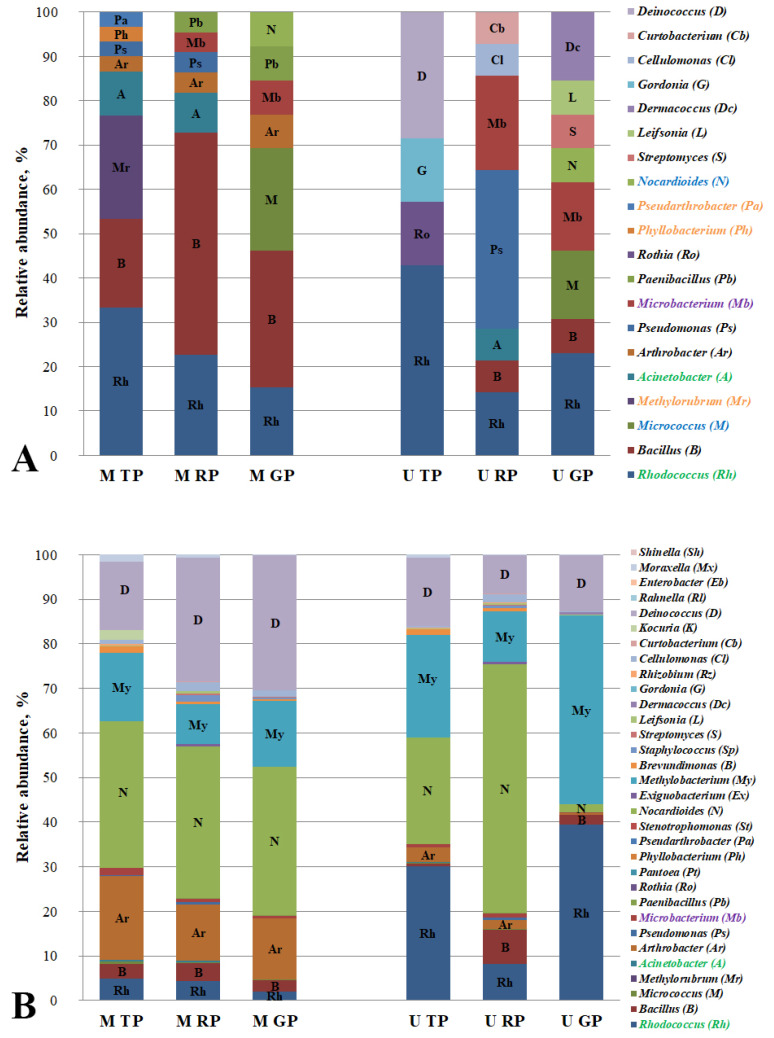
Relative abundance of isolated hydrocarbon-oxidizing bacteria (**A**) and these genera in microbiome (**B**) of road dust in Murmansk and Moscow functional zones. M TP, road dust from Moscow traffic zone; M RP, road dust from Moscow residential zone; M GP, road dust from Moscow recreational zone; U TP, road dust from Murmansk traffic zone; U RP, road dust from Murmansk residential zone; U GP, road dust from Murmansk recreational zone.

To date, extensive research has been carried out on degrading bacteria and the microbial communities of roadside soils and air [5,49]. Studies of road dust have mainly concentrated on its chemical composition and impacts, namely on the assessment of the amount and type of pollutants that are in the environment [6,7]. In the present work, in addition to assessing the chemical composition of the pollutants (PAHs), we also investigated the microbial community of the road dust, namely the PAH-degrading bacteria. The strains that were isolated in the course of the work belong to the following genera: *Rhodococcus*, *Bacillus*, *Micrococcus*, *Methylorubrum*, *Acinetobacter*, *Arthrobacter*, *Pseudomonas*, *Microbacterium*, *Paenibacillus*, *Rothia*, *Phyllobacterium*, *Pseudarthrobacter*, *Nocardioides*, *Streptomyces*, *Leifsonia*, *Dermacoccus*, *Cellulomonas*, *Curtobacterium*, and *Deinococcus*. An analysis of the relative abundance of the identified PAH-degrading microbial genera in the road-dust of the functional zones in Moscow and Murmansk is shown in Figure 2.

Bacteria of the genus *Arthrobacter* were found only in the road dust of all of the functional zones in Moscow. The strains of the genera *Phyllobacterium*, *Pseudarthrobacter,* and *Methylorubrum* were revealed only in the Moscow traffic zone (highlighted in orange in Figure 2A). An increase in the anthropogenic gradient was detected for the genera of the bacteria-degraders *Arthrobacter*, *Microbacterium,* and *Paenibacillus* in Moscow, and an increase was also seen in the gradient for *Bacillus* in Murmansk. It indicates the sensitivity of these road dust degraders to pollution. A negative trend in the anthropogenic gradient was observed for the samples from Moscow for *Rhodococcus* and *Acinetobacter*. It should be noted that the gradients of the Moscow functional zones for the isolated PAH-degraders were observed for the genera *Rhodococcus* and *Acinetobacter,* and their relative representation in the road dust microbiome have the same pattern (highlighted in green in Figure 2A,B), and for *Microbacterium*, the pattern of the gradients did not coincide (highlighted in lilac in Figure 2A,B). 

It has been emphasized that the pollution of the ambient air forms microbial leaf communities, thereby affecting the diversity of the supporting bacteria that are capable of degrading the airborne pollutants [50]. The air environment is the link between the two biotopes, the leaves and the paved surfaces, with respect to the spread of the chemical pollutant and the microbial communities’ members. A greater diversity of the hydrocarbon-oxidizing bacteria in both of the biotopes was found for the microbial community of the degraders that were isolated from the Murmansk dust samples. The quantitative taxonomic diversity of the PAH-degrading bacteria in the road dust in Moscow and Murmansk was equivalent; the primary differences between two cities concerned the microbial community of the degraders in the leaf dust. The genus diversity in the leaf dust from Murmansk was almost 2.5 times greater than the of the Moscow samples. When comparing the taxonomic diversity of the microbial destructors between the biotopes of each city, it should be noted that in Murmansk, we observed a smaller diversity of the genera in the road dust in contrast to that of the leaf dust, and in Moscow, a reverse but not significant trend was revealed.

Most of the microbial genera that were found in the course of this work are known to be active PAH degraders [4,51,52]. It should be noted that the ability to degrade the PAHs that were used in this work was observed for the genera *Rothia* and *Curtobacterium* for the first time. The hydrocarbon-oxidizing bacteria of the genus *Stenotrophomonas* (highlighted in blue in Figure 1A) were found only in the leaf dust of the traffic zones (highlighted in red in Figure 1A), and strains of the genera *Nocardioides* and *Micrococcus* were revealed to be only in the road dust of the recreational zones of Moscow and Murmansk (highlighted in blue in Figure 2A). Probably, the listed genera may be the representative PAH-degrading bacteria in certain biotopes and functional zones of the cities. Although a positive or negative trend with the anthropogenic pressures was observed in both of the cities, we found no common indicator of the PAH-degrading strain for all of the biotopes and urban functional zones that were studied (Figure S5). Probably, this can be related to the geographical location of the cities. However, in both Murmansk and Moscow, the *Micrococcus* PAH-degrading bacteria that were isolated from the leaf dust showed a positive gradient with the anthropogenic pressure. Therefore, we assume that the presence of the degrading strains of this genus in the microbiome can be considered as an indicator of the anthropogenic pollution of the plant phylloplane in the cities.

### 3.6. PAH of Road and Leaf Surfaces’ Dust of the Cities’ Functional Zones 

The spatial trends of the PAH total concentrations in the leaves that were observed in the urban areas indicate that there is a gradient of the PAH air pollution. Various authors [22,53,54,55] have observed the relationships between the degrees of PAH air pollution and the soil and/or leaf PAHs. In [56], the concentrations of phenanthrene, anthracene, and pyrene in the air along the roadsides of urbanized areas in Italy have been shown to be higher when they were compared to those of rural areas that were away from the road. In addition, the results of [57] show that phenanthrene is the most common PAH pollutant in the air; other common PAHs are fluoranthene, pyrene, naphthalene, and benzo[g,h,i]perylene.

**Figure 3:**
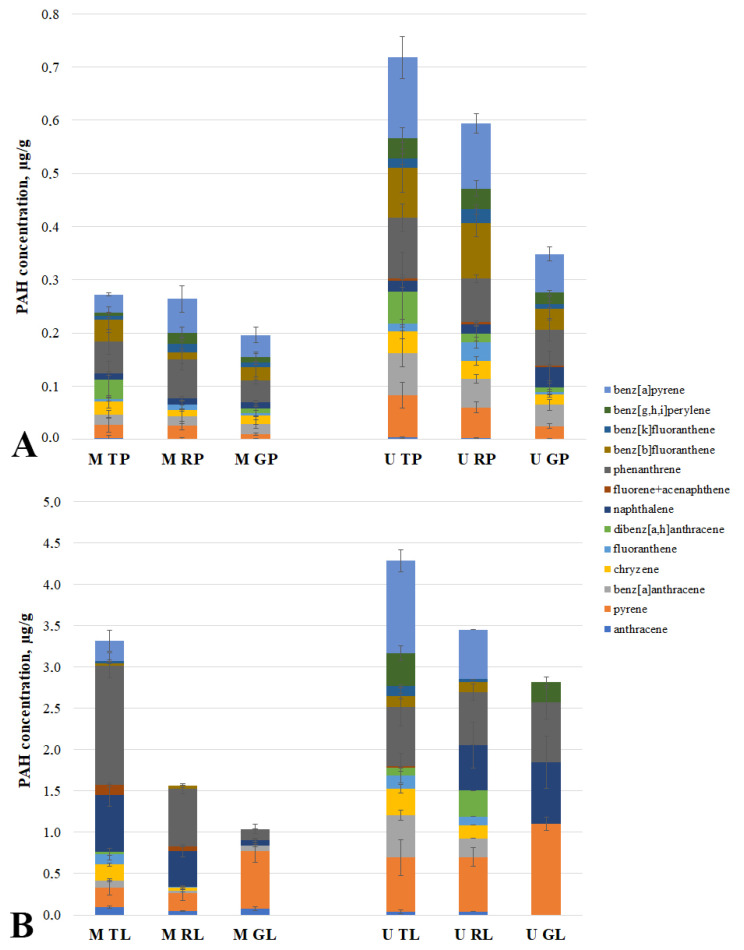
PAH concentration in road dust (**A**) and leaf dust (**B**) in Murmansk and Moscow functional zones. M TP, road dust from Moscow traffic zone; M RP, road dust from Moscow residential zone; M GP, road dust from Moscow recreational zone; U TP, road dust from Murmansk traffic zone; U RP, road dust from Murmansk residential zone; U GP, road dust from Murmansk recreational zone; M TL, leaf dust from Moscow traffic zone; M RL, leaf dust from Moscow residential zone; M GL, leaf dust from Moscow recreational zone; U TL, leaf dust from Murmansk traffic zone; U RL, leaf dust from Murmansk residential zone; U GL, leaf dust from Murmansk recreational zone.

In the course of the work, we identified the PAH-degrading bacteria within the gradient of the functional zones and the biotopes of Murmansk and Moscow. We were interested in testing for the presence of the corresponding gradients for the PAHs. Figure 3 shows the data on the concentrations of 13 PAHs for each of the studied sites.

The highest contents among the analyzed PAHs in the studied sites were observed for naphthalene, phenanthrene, benz[a]pyrene, and pyrene. The level of the road dust pollution was two times lower in Moscow than it was in Murmansk. At the same time, the level of the PAH pollution in the leaf dust in the traffic area of Moscow was different from that of the corresponding area in Murmansk. The total concentration of the PAHs in the leaf dust in the residential and recreational zones was two times higher in Murmansk than it was in Moscow. A negative gradient of the total PAH concentrations from the traffic zone to the recreational zone of Murmansk and Moscow was found only in the leaf surface dust. A similar effect for the road dust was observed only for the functional zones of Murmansk. In the leaf dust of both of the cities, a negative trend was observed with the decrease in the anthropogenic load with respect to the benzo[a]pyrene concentrations. A similar gradient was also revealed for phenanthrene, naphthalene, and chrysene in the leaf dust in Moscow, and for benzanthracene and chrysene in Murmansk. In the dust from the road of both of the cities, a negative trend was observed with the decrease in the anthropogenic load for the pyrene concentrations. In addition, in Murmansk, the effect for this biotope was similar for those of benzo[a]pyrene, phenanthrene, and dibenz[a,h]anthracene. In summarizing the results that were obtained, we can confirm the data of other studies [7,52,58] that demonstrate that there is a decrease in the anthropogenic environmental pollution depending on the distance from the pollution source.

### 3.7. Correlation of the Studied Characteristics of Functional Zones Biotopes 

**Figure 4:**
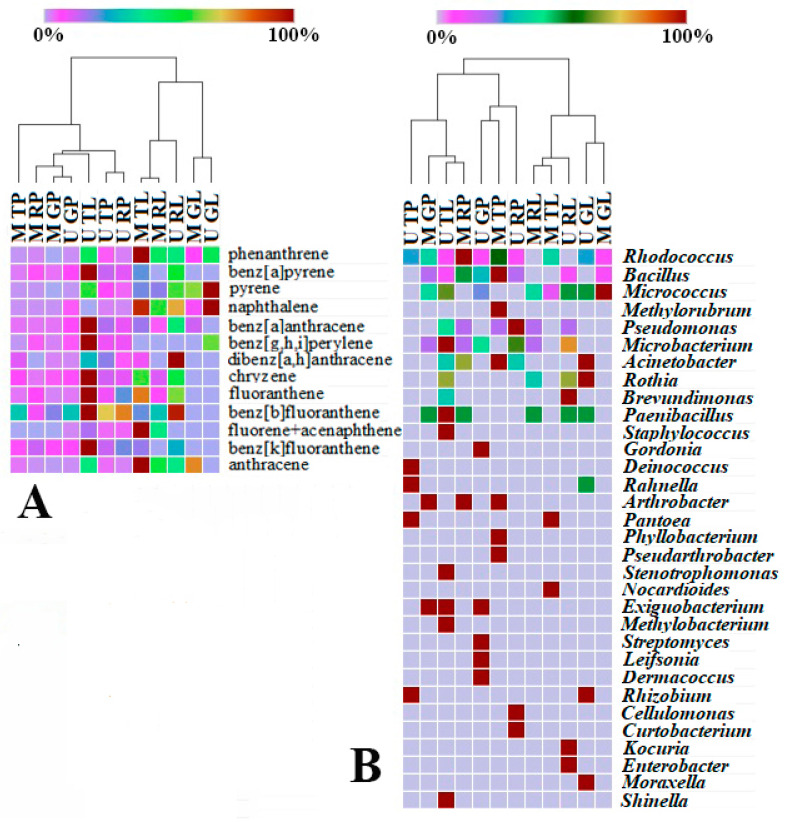
Heat maps of the PAHs (**A**) and genera of hydrocarbon-oxidizing bacteria in microbiome (**B**) in relation to Murmansk and Moscow functional zones and biotopes. M TP, road dust from Moscow traffic zone; M RP, road dust from Moscow residential zone; M GP, road dust from Moscow recreational zone; U TP, road dust from Murmansk traffic zone; U RP, road dust from Murmansk residential zone; U GP, road dust from Murmansk recreational zone; M TL, leaf dust from Moscow traffic zone; M RL, leaf dust from Moscow residential zone; M GL, leaf dust from Moscow recreational zone; U TL, leaf dust from Murmansk traffic zone; U RL, leaf dust from Murmansk residential zone; U GL, leaf dust from Murmansk recreational zone. Color code from blue (0%) to brown (100%) depicts relative amount of respective group (PAHs or genera of bacteria).

It has been demonstrated that the number of hydrocarbon-degrading bacteria on evergreen leaves increases with increasing hydrocarbon concentrations [10]. The authors have suggested that when the PAHs are deposited in high concentrations on the leaves, they are biodegraded in the phyllosphere, which contributes to urban air cleansing. In contrast to the cited work, our study compared two biotopes of three functional zones in two cities with different climates. To check for a correlation between the data that were obtained in the work on the diversity of the PAH-degrading bacteria and the quantitative and qualitative content of the dust, we performed the Pearson correlation analysis. The results of correlation analyses between the biotopes of the functional zones of the two cities and the quantitative and qualitative composition of the PAHs, as well as between the biotopes of the functional zones of the two cities and the relative abundance at the genus level of the isolated microbial degraders are given in Figure 4.

The heat map of the relative content and diversity of the PAHs in the dust samples from the road and leaves of various urban functional zones of Moscow and Murmansk demonstrates that the data that were obtained are clustered into two large groups, one of which refers to the data of the samples that are exclusively of the “leaf surfaces” biotope of various zones in both of the cities. The second large cluster includes the samples of the “road” biotope with the exception of a sample of leaf dust from the Murmansk traffic zone (U TL). This is clustered into a separate subgroup. The sample (U TL) is characterized by it having the highest total content and diversity of the composition of the studied PAHs in comparison with that of the other samples of the dust from the leaf surfaces. This is indicative of a strong pollution of atmospheric air and, consequently, of both of the biotopes in the traffic zone of Murmansk. Probably, such a strong pollution of the leaves is because the dust sampling point in the traffic zone is located in the immediate vicinity of the Murmansk Thermal power station [59,60].

It should be noted that the leaf dust samples from the three functional zones of Murmansk (U TL, U RL, and U GL) vary greatly in their composition of PAHs and belong to three different clusters, with the traffic zone being the most polluted. In the case of the leaf dust samples from Moscow, the pattern is different: the dust from the traffic zone (M TL) and the residential zone (M RL) belong to the same subgroup, and the samples from the recreational zone (M GL) are attributed to another cluster. Moreover, the dust samples from the roads of the recreational areas of Moscow and Murmansk (M GP and U GP) are allocated to one subgroup, as are the dust samples from the leaves (M GL and U GL). This suggests that there is a similar pattern regarding the PAH pollution in the recreational areas of Murmansk and Moscow.

The hierarchical clusters of both the PAH concentration (Figure 4A) and the relative representation of the isolated PAH degraders (Figure 4B) in the biotopes of the cities were divided into two large clusters. In two heat maps, a sample of the dust from the leaves of the Murmansk traffic zone (U TL) was part of a cluster with the dust samples from the road. This sample is similar in the distribution of its degraders to the dust sample from the road of the residential area of Moscow (M RP). Although the road dust samples from the recreational zones of Moscow and Murmansk (M GP and U GP) in Figure 4A are assigned to the same subgroup, their corresponding degraders differed greatly (Figure 4B). It suggests that the formation of and change in the composition of the degraders’ communities in the road dust from the recreational zones are influenced not only by the qualitative and quantitative composition of the pollutants, but also by other factors, likely climatic conditions and the absence of the intensive transfer of bacteria due to the dust resuspension that is induced by the movement of cars [37]. This makes the microbial communities of the road dust from the recreational areas more specific, unlike the communities of the dust from the leaves, where one of the main factors influencing the similarity of the microbiome compositions is the type of plant.

To detect the relationship between the isolated degraders, the studied PAHs, and the biotopes of the functional zones of the cities, a PCA and a linear regression were used.

**Figure 5:**
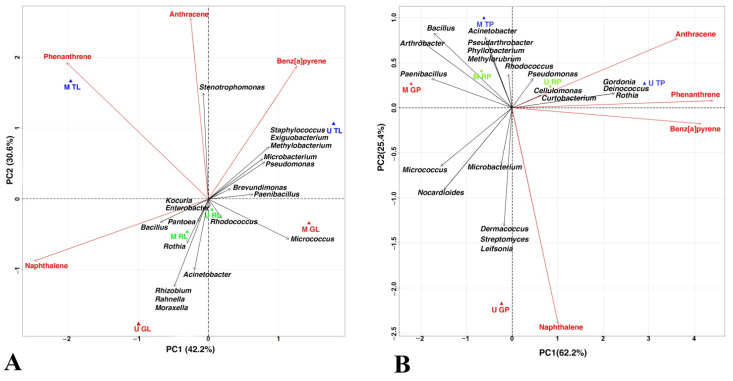
Principal Component Analysis of the selected elemental components detected in the two biotopes: (**A**) dust from leaf surfaces and (**B**) road dust at the three sampling sites in Moscow and Murmansk (*p* < 0.05).

The first two axes together describe 72.8% and 87.6% of total variance for the leaf dust and the road dust, respectively (Figure 5). In the case of the leaf dust, PC1 was positively correlated with the *Paenibacillus* content, and it was negatively correlated with naphthalene (Figure 5A). PC2 was positively correlated with the anthracene and *Stenotrophomonas* content, and it was negatively associated with *Acinetobacter*. The traffic zones are grouped on the positive side of the PC2 graph. Phenanthrene was positively correlated with *Stenotrophomonas,* and it was negatively correlated with *Micrococcus*. Anthracene was positively associated with *Stenotrophomonas,* and it was negatively associated with *Acinetobacter*. A positive correlation with *Bacillus* and negative correlation with *Pseudomonas* were found for naphthalene. Benz[a]pyrene was positively associated with *Exiguobacterium, Methylobacterium, Microbacterium,* and *Stenotrophomonas,* and it was negatively associated with *Rothia*.

In the case of the road dust, PC1 was positively correlated with the content of phenanthrene, benz[a]pyrene, and *Rothia*, and it was negatively correlated with *Paenibacillus* (Figure 5B). PC2 was positively associated with content of the *Rhodococcus, Acinethobacter,* and *Methylorubrum,* and it was negatively correlated with *Microbacterium, Streptomyces, Dermacoccus,* and *Leifsonia*. The localization of U GP indicates its difference from the road dust biotopes of the other functional zones of the studied cities. All of the zones of Moscow are located on the left side of PC1. A positive correlation with *Microbacterium, Streptomyces, Dermacoccus,* and *Leifsonia* for naphthalene was found, and a negative association with *Pseudarthrobacter, Phyllobacterium, Acinethobacter,* and *Methylorubrum* for naphthalene was found. Phenanthrene was positively correlated with *Rothia, Gordonia,* and *Deinococcus*. Anthracene was positively associated *Rothia, Gordonia, Pseudomonas,* and *Deinococcus,* and it was negatively correlated with *Micrococcus*. Benz[a]pyrene was negatively associated with *Paenibacillus*.

A detailed stepwise linear regression analysis was used to explain the patterns that were obtained by the PCA. The obtained data confirmed that *Micrococcus* was sensitive to the anthropogenic pollution of the urban ecosystems. The contribution of the other studied components were not significant.

The obtained results indicate, once again, that there is a multifactorial effect of various anthropogenic pollutants on the ecosystem as a whole and its microbial community, in particular. The individual biotopes of the “clean” recreational zones of the two cities are clearly similar, probably because the park areas are less exposed to anthropogenic pollution in comparison with the other functional zones that were studied.

It should be noted that the microbiomes that were measured using static methods such as assessing the diversity of the microbial community based on the sequencing of the marker gene sequences included the entire microbial biomass, and therefore, this does not allow it to be divided into the active and inactive biomass [61]. At the same time, the inactive biomass can make up a large part of the microbiome [62]. For example, in soil, the active bacteria account for only 0.1–2% of the microbial biomass, and the potentially active bacteria account for 10–40% of this [63]. Therefore, to assess the functional degradative properties of the microbial community, the classical method of microbiology in combination with the taxonomic profiling of the microbial community and the chemical composition of the PAHs were used.

The sensitivity of the degraders to pollution suggests their possible application as a tool for monitoring the anthropogenic changes in the biosphere, for example, for the early diagnosis of the technogenic damages to the pedosphere, phylloplane, and atmosphere. For example, some of the features in the structure of the prokaryotic communities of the degraders that were isolated from the leaf dust revealed the sensitive to qualitative and quantitative composition of the PAHs, and these can be used as bioindicators of the environmental quality. The obtained results expand our understanding of the distribution of the PAH-degraders in technogenically disturbed zones and can provide an input for the creation of an effective biomonitoring system, thereby contributing to the sustainable development of the urban environment.

The changes in the quantitative and qualitative composition of the microbial communities that are associated with the PAHs are dependent on the distance between them, the pollution source, and the sampling season, and this was confirmed to be true for different climatic regions and biotopes [6,25,37,44]. Comprehensive studies in industrial cities across climatic zones have contributed to the expansion of our knowledge about the role of these bacteria in the processes of removing the pollutants from the ecological systems. Furthermore, this will make it possible to understand whether there are degrader groups that are common to all of the climatic zones, or whether there is a certain degrader cluster in each region which is formed under specific conditions.

## 4. Conclusions

To sum up, the samples that were collected in the functional zones of Murmansk were found to have 27 genera of the PAH-degrading bacteria; in Moscow, there were 15. It appears that Murmansk features multifactor pollution, and as a result, the community of the PAH degraders from the surface of the leaves had a greater similarity with those in road dust samples in contrast to those in Moscow. 

A lower anthropogenic load determined that there is a similarity in the PAH pollution and diversity of the PAH-degrading bacteria in different the biotopes of Moscow’s and Murmansk’s recreational zones. The microbial communities of the PAH degraders in the leaf dust samples differed from the microbiomes in the road dust. In addition, when analyzing the data from the leaf biotope, we observed that there were large differences in the PAH distribution when this was compared to that of the road biotope. This confirms that leaves are an indicator of air pollution in large cities [47].

The PAH-degrading bacteria of the genus *Micrococcus* proved to be sensitive to the pollution of the ecosystems with PAHs. Despite their ability to oxidize PAHs, additional factors (nutrients, trace elements, etc.) are apparently present in the green zone biotopes which contribute to a higher relative abundance of these degraders’ genera.

The data that were obtained suggest that the degrading strains of the genera which were identified in this work are minor representatives of these genera in the microbial community, and they may play key roles in PAH degradation. These results expand our views of the PAH degraders’ distribution in different functional urban zones. The high indicative capacity of the degrading bacteria of the genus *Micrococcus*, based on their relationship to the level of the PAH pollution in the ecosystems, enables researchers to choose them as a tool for monitoring the anthropogenic changes in the biosphere.

The logical difficulties that are caused by the constant change in the intensity and characteristics of the technogenic load, the variation in the size and configuration of negative anthropogenic influence zones, and the management and natural changes in the phytocenoses and soils as a result of urban redevelopment arise when one is monitoring the distribution and accumulation of the organic pollutants in natural objects under urban conditions. An important aspect of monitoring the urban ecosystem is the assessment of the PAHs’ impact on the changes in the microbial communities of various biotopes. Bacteria with a huge variety of enzyme systems and high lability of their metabolism are mainly responsible for the self-purification of natural ecosystems, including urban ecosystems, and they are capable of carrying out the biodegradation of natural and synthetic xenobiotics, thereby returning the main nutrients to the global cycle. This research is a groundwork for understanding the extent to which the microbial community of a particular biotope has a pool of degrading bacteria that contribute to the purification of the system. Seasonal long-term studies would be required to deepen our knowledge of the capacity of the urban ecosystems for the self-cleaning.

## Figures and Tables

**Table 1:** List of hydrocarbon-oxidizing bacteria isolated from dust that was collected from road and leaf surfaces.

Genus	Functional Zone	Biotope	Strain	Substrate Specificity *
Moscow				
*Acinetobacter*		Road dust	M TP4a-1BP	Phn, Sal, BaP, Nah
	Traffic		M TP75aPH	Phn, Ant, Sal, BaP, Nah
		Leaf dust	M TL4PH-2	Phn, Sal, BaP, Nah
	Residential	Road dust	M RP4b-2BP	BP, Nah
		Leaf dust	M RL4a-1BP	Phn, Sal, BaP, Nah
*Arthrobacter*	Traffic		M TP19A	Phn, Ant, BaP, Nah
	Residential	Road dust	M RP23-2PH	Phn, Sal, BaP, Nah
	Recreational		M GP3PH	Phn, Ant, Sal, BaP, Nah
*Bacillus*			M TP75bPH	Phn, Ant, Sal, BaP, Nah
			M TP12A	Phn, Ant, Sal, BaP, Nah
			M TP24N	Phn, Ant, Sal, BaP, Nah
			M TP4BP	Phn, Sal, BaP, Nah
		Road dust	M TP2BP-3	Sal, BaP
	Traffic		M TP2BP-2	BaP
			M TP21A	Phn, Ant, Nah
			M TP5A	Phn, Ant, BaP, Nah
		Leaf dust	M TL38N2	Phn, Sal, BaP, Nah
			M TL36N-2	BaP, Nah
			M RP5N	Ant, Sal, BaP, Nah
			M RP1PH	Phn, Sal, BaP, Nah
			M RP60N2	Phn, Ant, BaP, Nah
			M RP8PH	Phn, Ant, Sal, BaP, Nah
		Road dust	M RP11PH	Phn, Ant, Sal, BaP, Nah
			M RP16-2PH	Phn, Ant, Sal, BaP, Nah
	Residential		M RP16-1BP	Phn, Ant, Sal, BaP, Nah
			M RP22PH	Phn, Ant, Sal, BaP, Nah
			M RP17PH	Phn, Ant, Sal, BaP, Nah
			M RP9PH	Phn, Ant, Sal, Nah
		Leaf dust	M RL23-1PH	Phn, Nah
			M RL4aBP	Phn, BaP, Nah
		Road dust	M GP47PH	Phn, Ant, Sal, Nah
			M GP54PH	Phn, Ant, Sal, BaP, Nah
	Recreational		M GP21N	Phn, Ant, Sal, BaP, Nah
			M GP37N	Phn, Ant, Sal, BaP, Nah
		Leaf dust	M GL3N	Phn, BaP, Nah
*Methylorubrum*			M TP31BP-2	Ant, BaP
			M TP4BP-2	Ant, Sal, BaP
	Traffic	Road dust	M TP6BP-2	Ant, Sal, BaP
			M TP1BP-2	Ant, Sal, BaP
			M TP3BP-2	Sal, BaP
		Leaf dust	M TL33BP-2	Phn, Sal, BaP, Nah
*Microbacterium*	Traffic	Road dust	M RP4b-1BP	Sal, BaP, Nah
	Residential	Road dust	M GP1a-2BP	BaP, Nah
*Micrococcus*	Traffic	Leaf dust	M TL5BP-2	Phn, Sal, BaP, Nah
			M RL3BP-2	BaP
	Residential	Leaf dust	M RL1BP-2	Sal, BaP
			M RL5BP-2	Sal, BaP
			M GP4BP	Ant, Sal, BaP
		Road dust	M GP9BP	Ant, Sal, BaP
			M GP8BP	Phn, Ant, Sal, BaP, Nah
	Recreational		M GL1A-1BP	Sal, BaP
			M GL5N	Sal, Nah
			M GL1PH	Phn, Sal, BaP, Nah
		Leaf dust	M GL4N	Phn, Sal, BaP, Nah
			M GL7PH	Phn, Sal, BaP, Nah
			M GL3aBP	Phn, Sal, BaP, Nah
			M GL6N	Phn, Sal, BaP, Nah
			M GL8N	Phn, Sal, BaP, Nah
*Nocardioides*	Recreational	Road dust	M GP7BP	BaP
*Pantoea*	Residential	Road dust	M RP2-1N	Sal, BaP, Nah
*Paenibacillus*	Recreational	Road dust	M RP27PH1	Phn, Ant, BaP
	Recreational	Road dust	M GP44PH	Phn, Ant, BaP, Nah
*Phyllobacterium*	Traffic	Road dust	M TP10BP	Phn, Ant, BaP
*Pseudarthrobacter*	Traffic	Road dust	M TP3PH	Phn, Ant, Sal, BaP, Nah
*Pseudomonas*	Traffic	Road dust	M TP2PH	Phn, Ant, BaP, Nah
		Leaf dust	M TL29N	Phn, Sal, BaP, Nah
	Recreational	Road dust	M RP6PH	Phn, Ant, Sal, BaP, Nah
			M TP11BP	Ant, Sal, BaP, Nah
			M TP1BP	Ant, Sal, BaP, Nah
			M TP5BP	Sal, BaP, Nah
			M TP8BP	Sal, BaP, Nah
	Traffic	Road dust	M TP5PH	Phn, Ant, Sal, BaP, Nah
			M TP2BP	Phn, Ant, Sal, BaP, Nah
			M TP9BP	Phn, Ant, Sal, BaP, Nah
			M TP13BP	Phn, Sal, BaP, Nah
*Rhodococcus*			M TP14BP	Phn, Ant, BaP, Nah
			M TP7-2BP	Ant, BaP
			M RP1b-1BP	Sal, BaP
			M RP3BP	Ant, Sal, BaP, Nah
	Residential	Road dust	M RP2B-2BP	Ant, Sal, BaP, Nah
			M RP2N1	Sal, Nah
			M RP15PH	Phn, Ant, Sal, BaP, Nah
		Road dust	M GP45bPH	Phn, Ant, Sal, BaP, Nah
	Recreational		M GP35A	Phn, Ant, BaP, Nah
		Leaf dust	M GL2PH	Phn, Sal, BaP, Nah
*Rothia*	Traffic	Leaf dust	M TL3PH-2	Phn, Sal, BaP, Nah
	Residential	Road dust	M RP4a-2BP	Ant, Sal, BaP, Nah
*Stenotrophomonas*	Traffic	Road dust	M TP3aBP	Phn, Ant, BaP, Nah
Murmansk				
	Traffic	Leaf dust	U TL4BP	Phn, Ant, Sal, BaP, Nah
			U TLBP4-1	Phn, Ant, Sal, BaP, Nah
		Road dust	U RPPH3	Phn, Ant, Sal, BaP, Nah
	Residential		U RLBP5-2	Phn, BaP
*Acinetobacter*		Leaf dust	U RLBP1-2	Phn, Ant, BaP
			U RLBP3-2	Ant, BaP
			U GLBP4-2	Phn, Ant, Sal, BaP, Nah
	Recreational	Leaf dust	U GLN2-2	Phn, Ant, Sal, BaP, Nah
			U GLN3-1	Sal, BaP, Nah
	Traffic	Leaf dust	U TLA4	Ant, Sal, BaP, Nah
	Residential	Road dust	U RPN1-2	Phn, Ant, BaP, Nah
*Bacillus*		Road dust	U GPPH1-2	Phn, Sal, BaP, Nah
	Recreational		U GLBP6	Phn, Ant, Sal, BaP, Nah
		Leaf dust	U GLPH1-2	Phn, Ant, Sal, BaP, Nah
			U GLBP4	Phn, Ant, Sal, BaP
*Brevundimonas*	Traffic	Leaf dust	U TLBP4-1	Phn, Ant, Sal, BaP, Nah
			U RLBP5-2	Ant, BaP
	Residential	Leaf dust	U RLBP1-2	Phn, Ant, BaP
			U RLBP3-2	Ant, BaP
*Cellulomonas*	Residential	Road dust	U RPBP1	Ant, BaP, Nah
*Curtobacterium*	Residential	Road dust	U RPN1-1	Phn, Sal, Nah
*Deinococcus*	Traffic	Road dust	U TPPH2	Phn, Ant, Sal, BaP
			U TPA1	Phn, Ant, Sal, BaP, Nah
*Dermacoccus*	Traffic	Road dust	U GPN1	Phn, Ant, BaP, Nah
			U GPN3	Phn, Ant, BaP, Nah
*Exiguobacterium*	Traffic	Leaf dust	U TL1BP	Phn, Ant, BaP
*Gordonia*	Traffic	Road dust	U TPA4-2	Phn, Ant, BaP
*Kocuria*	Residential	Leaf dust	U RLPH3	Phn, Sal, Nah
*Leifsonia*	Recreational	Road dust	U GPA3	Ant, Sal, BaP
*Enterobacter*	Residential	Leaf dust	U RLBP5	Phn, Ant, Sal, BaP
*Methylobacterium*	Traffic	Leaf dust	U TLPH5	Phn, Ant
			U TLPH2-2,	Phn, Sal, BaP, Nah
			U TLPH5-1	Phn, Sal, BaP, Nah
	Traffic	Leaf dust	U TLBP5-2	Phn, Ant, Sal, BaP, Nah
			U TLPH6-2	Phn, Ant, Sal, BaP
*Microbacterium*			U TLPH6	Phn, Ant, Sal, BaP
			U RPN2	Sal, BaP, Nah
		Road dust	U RPPH4	Phn, BaP, Nah
			U RPBP3	Ant, Sal, BaP, Nah
	Residential		U RLBP1	BaP
		Leaf dust	U RLN2	Phn, Sal, Nah
			U RLBP2	BaP, Nah
			U RLA2	Ant
	Recreational	Road dust	U GPBP5	Phn, Ant, BaP, Nah
			U GPPH4	Phn, Ant, Sal, BaP
			U TLBP2	Phn, Ant, Sal, BaP, Nah
			U TLPH4-1	Phn, Ant, Sal, BaP, Nah
	Traffic	Leaf dust	U TLPH8	Phn, Ant, Sal, BaP, Nah
			U TL6BP	Phn, Ant, Sal, BaP, Nah
			U TL7BP	Phn, Ant, Sal, BaP, Nah
		Road dust	U RPPH3-2	Phn, Ant, Sal, BaP, Nah
*Micrococcus*	Residential		U RLPH1	Phn, Ant, Sal, BaP, Nah
		Leaf dust	U RLN3	Ant, BaP, Nah
			U RLBP1	Ant, Sal, BaP, Nah
		Road dust	U GPA3-2,	Ant, Sal, BaP, Nah
			U GPPH4-2	Phn, Sal, BaP, Nah
	Recreational		U GLN2	Phn, Ant, Sal, BaP, Nah
		Leaf dust	U GLN2-1	Phn, Ant, Sal, BaP, Nah
			U GLN3	Phn, Ant, Sal, BaP, Nah
			U GLPH1	Phn, Ant, BaP
*Moraxella*	Recreational	Leaf dust	U GLA1	Phn, Ant, BaP
*Nocardioides*	Recreational	Road dust	U GP6BP	BaP, Nah
	Traffic	Leaf dust	U TLA2	Ant, Sal, BaP, Nah
*Paenibacillus*			U TLPH1	Phn, Ant, Sal, BaP, Nah
	Residential	Road dust	U RLPH1-1	Phn, Ant, Sal, BaP, Nah
	Recreational	Leaf dust	U GLN7-2	Ant, Sal, BaP, Nah
	Traffic	Leaf dust	U TLPH1-1	Phn, BaP, Nah
			U TLPH2	Phn, Ant, BaP, Nah
			U RPPH2,	Phn, Sal, BaP, Nah
*Pseudomonas*			U RPPH1	Phn, Sal, BaP, Nah
	Residential	Road dust	U RPPH4-2	Phn, BaP, Nah
			U RPN1	Ant, Sal, Nah
			U RPN3	Sal, BaP, Nah
*Rahnella*	Recreational	Leaf dust	U GLA2	Ant, Nah
*Rhizobium*	Recreational	Leaf dust	U GLPH2-2	Phn, Ant
			U TPPH5	Phn, Sal, BaP, Nah
	Traffic	Road dust	U TPN1	Ant, Sal, BaP, Nah
			U TPPH7	Phn, Ant, Sal, BaP, Nah
		Leaf dust	U TLN2	Ant, Sal, BaP, Nah
			U RPPH2-2	Phn, Ant, Sal, BaP, Nah
*Rhodococcus*	Residential		U RPPH1-2	Phn, Ant, Sal, BaP, Nah
		Leaf dust	U RLBP3	Phn, Ant, Sal, BaP, Nah
			U GPBP1	Phn, BaP, Nah
	Recreational	Road dust	U GPBP3	Ant, BaP, Nah
			U GPN2	Phn, Ant, Sal, BaP, Nah
*Rothia*		Road dust	U TPA4	Phn, Ant, Sal, BaP, Nah
	Traffic	Leaf dust	U TLBP5-1	Phn, Ant, BaP, Nah
			U TL3-1BP	Phn, Ant, BaP, Nah
	Residential	Leaf dust	U RLN1	Phn, Ant, BaP, Nah
			U RLBP1-1	Phn, Ant, BaP, Nah
			U GLPH2	Phn, Ant, BaP, Nah
	Recreational	Leaf dust	U GLBP5	Phn, Ant, BaP, Nah
			U GLN1	Ant, BaP, Nah
*Shinella*	Traffic	Leaf dust	U TL3BP	BaP
*Staphylococcus*	Traffic	Leaf dust	U TLPH1-2	Phn, Sal
			U TLA3-2	Ant, Sal, BaP
*Stenotrophomonas*	Traffic	Leaf dust	U TLBP4	Phn, BaP
*Streptomyces*	Recreational	Road dust	U GPA1	Phn, Ant, Sal, BaP

* Phn, phenanthrene; Ant, anthracene; Sal, salicylate; BaP, benzo[a]pyrene; Nah, naphthalene.

## Data Availability

All data is reported in this article.

## Supplementary Materials

The following are available online at https://www.mdpi.com/article/10.3390/microorganisms10101979/s1, **Figure S1:** Meteorological condition in Moscow (A) and Murmansk (B) in the summer of 2021 are two weeks prior to the sampling, **Figure S2:** Overview of the three urban sites (Traffic, Residential and Recreational zones) of Moscow (A) and Murmansk (B), **Figure S3:** Areal density of collected dust from road and leaf surfaces. M—Moscow, U—Murmansk. TL—leaf dust collected in traffic zone, RL—leaf dust collected in residential zone, GL—leaf dust collected in recreational zone, **Figure S4:** Amount of microorganisms of Murmansk (U) and Moscow (M) biotopes dust. TL—leaf dust collected in traffic zone, RL—leaf dust collected in residential zone, GL—leaf dust collected in recreational zone, **Figure S5:** Venn diagram of common genera of bacteria-degraders found in biotopes (I) and functional zones (II) of Moscow and Murmansk. M TL—leaf dust from Moscow traffic zone, M RL—leaf dust from Moscow residential zone, M GL—leaf dust from Moscow recreational zone, U TL—leaf dust from Murmansk traffic zone, U RL—leaf dust from Murmansk residential zone, U GL—leaf dust from Murmansk recreational zone. Coincidences of community membership between the studied sites are indicated by overlap in the diagram, **Figure S6:** Phylogenetic trees of strains-degraders isolated from road and leaf dust of Moscow (A) and Murmansk (B), computed by PyElph software using Neighbor Joining method. Red rectangles indicate closely related strains-degraders isolated from different biotopes or functional zones, **Figure S7:** Linear regression analysis between normalized PAH (Ant-anthracene and Phn-phenanthrene) and normalized amount of *Micrococcus* degrader in the three urban sites of Moscow and Murmansk of leaf (blue) and road (orange) dust. Statistical significance of R-squared coefficient was reported (*p* < 0.05), **Table S1:** The range of the total relative abundance of the PAH-degrading genera.
