# Supplementary material for: Polycyclic Aromatic Hydrocarbon-Degrading Bacteria in Three Different Functional Zones of the Cities of Moscow and Murmansk
Source: Microorganisms. 2022 Oct 6;10(10):1979. doi: 10.3390/microorganisms10101979 (PMC9610461; doi:10.3390/microorganisms10101979)
Supplement: Supplementary file 1 [file microorganisms-10-01979-s001.zip › Supplementary material.pdf]

Supplementary material

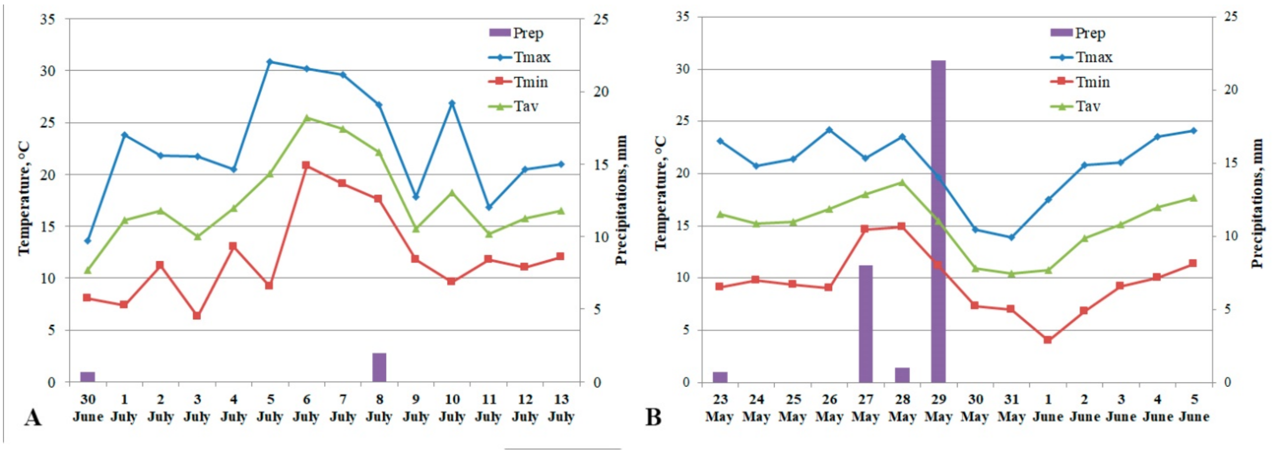

Figure S1. Meteorological condition in Moscow (A) and Murmansk (B) in the summer of 2021 are two weeks prior to the sampling.

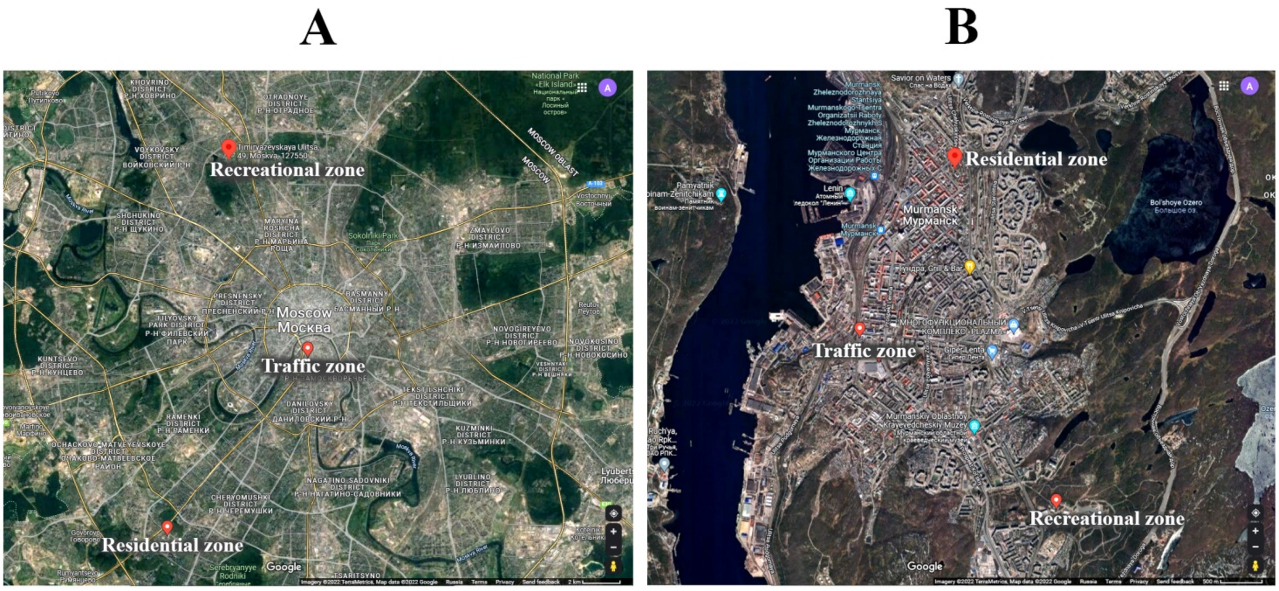

Figure S2. Overview of the three urban sites (Traffic, Residential and Recreational zones) of Moscow (A) and Murmansk (B).

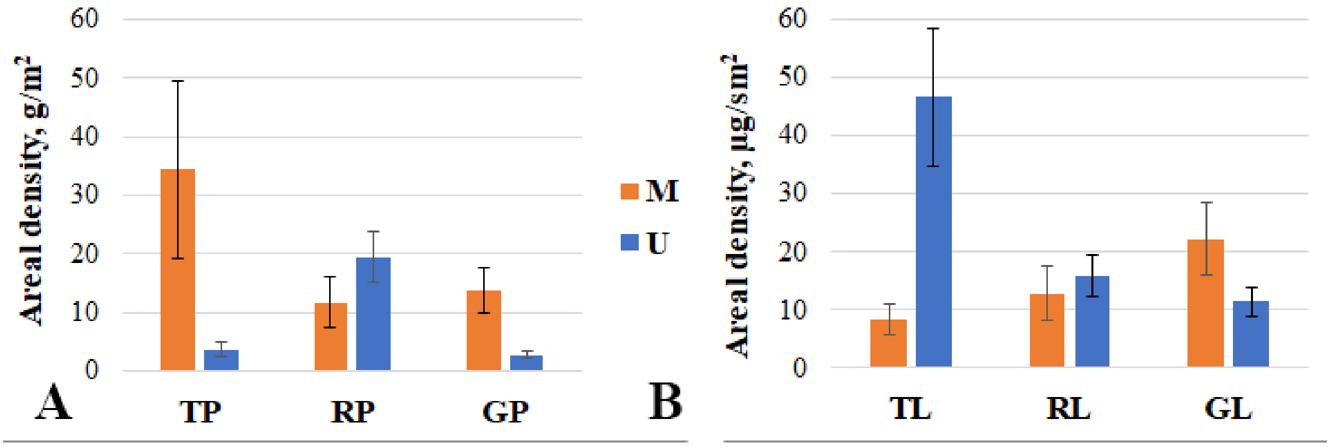

Figure S3. Areal density of collected dust from road and leaf surfaces. M – Moscow, U – Murmansk. TL – leaf dust collected in traffic zone, RL – leaf dust collected in residential zone, GL – leaf dust collected in recreational zone.

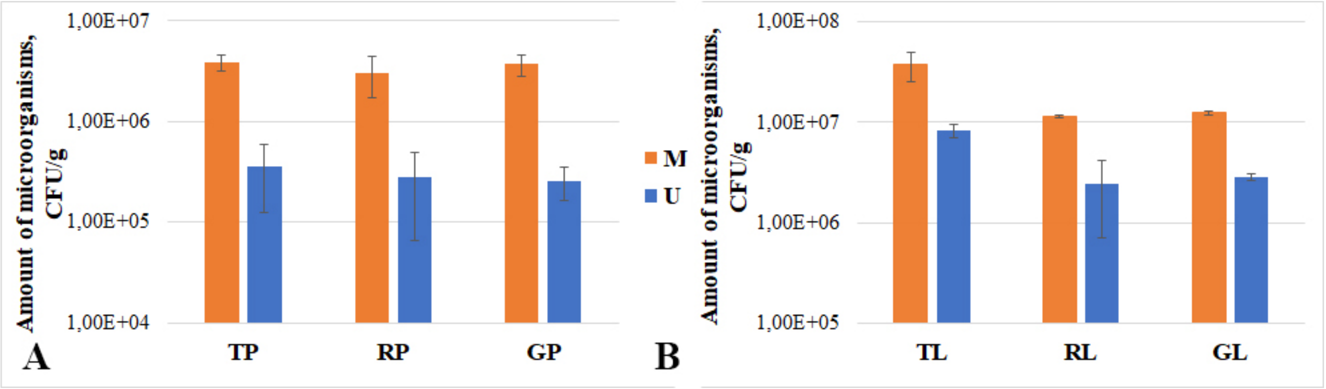

Figure S4. Amount of microorganisms of Murmansk (U) and Moscow (M) biotopes dust. TL – leaf dust collected in traffic zone, RL – leaf dust collected in residential zone, GL – leaf dust collected in recreational zone.

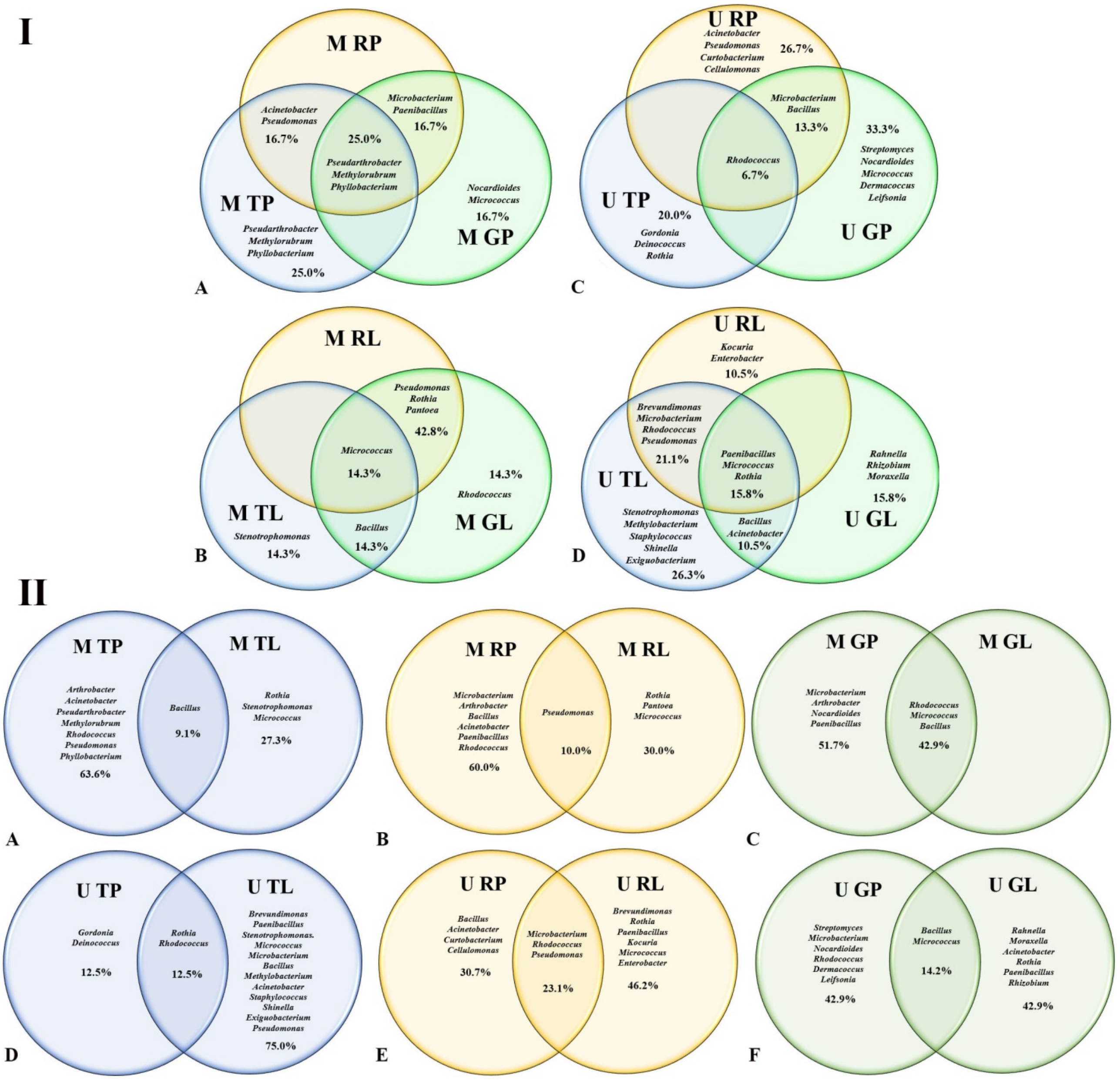

Figure S5. Venn diagram of common genera of bacteria-degraders found in biotopes (I) and functional zones (II) of Moscow and Murmansk. M TL – leaf dust from Moscow traffic zone, M RL – leaf dust from Moscow residential zone, M GL – leaf dust from Moscow recreational zone, U TL – leaf dust from Murmansk traffic zone, U RL – leaf dust from Murmansk residential zone, U GL – leaf dust from Murmansk recreational zone. Coincidences of community membership between the studied sites are indicated by overlap in the diagram.

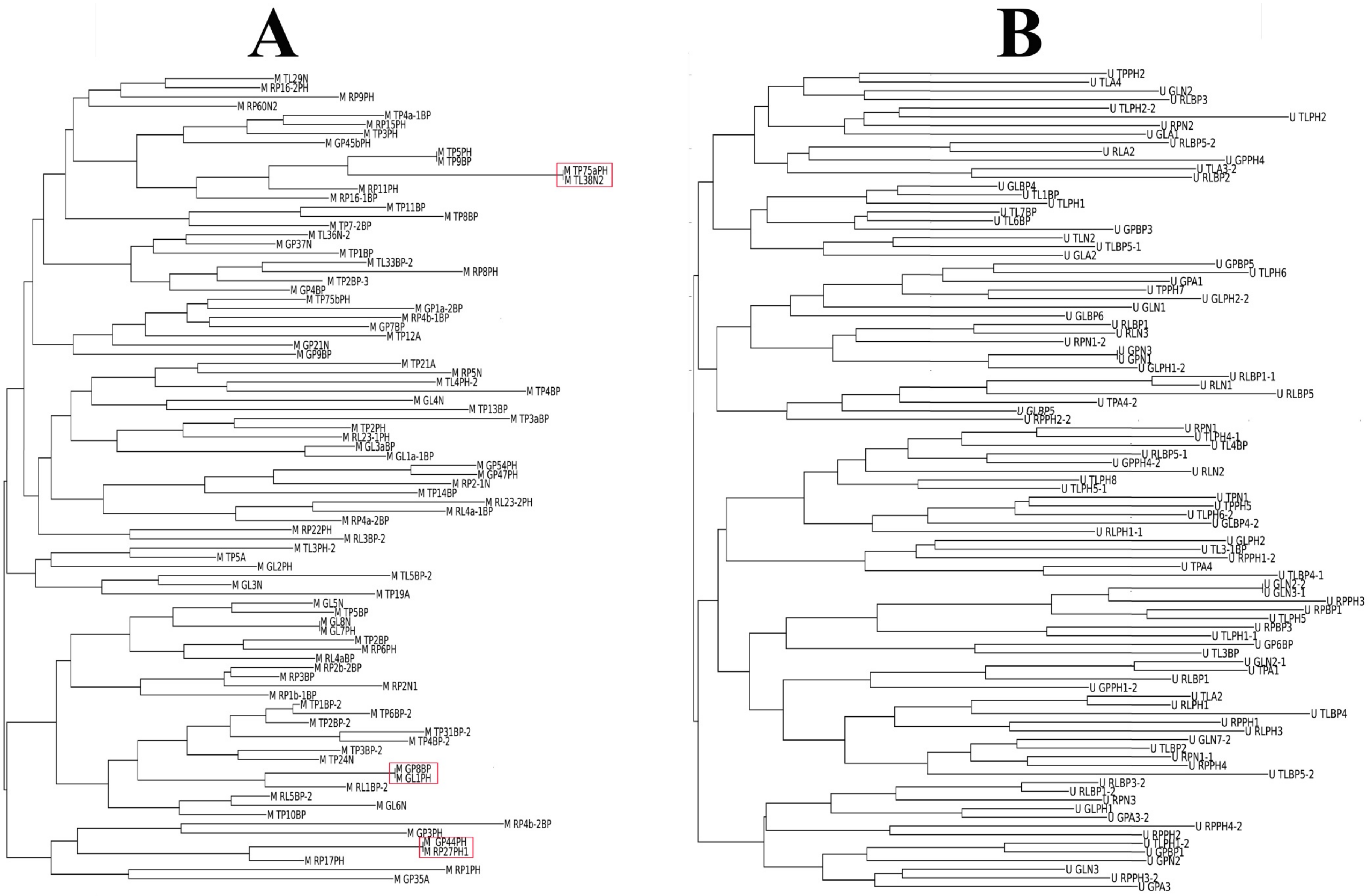

Figure S6. Phylogenetic trees of strains-degraders isolated from road and leaf dust of Moscow (A) and Murmansk (B), computed by PyElph software using Neighbor Joining method. Red rectangles indicate closely related strains-degraders isolated from different biotopes or functional zones.

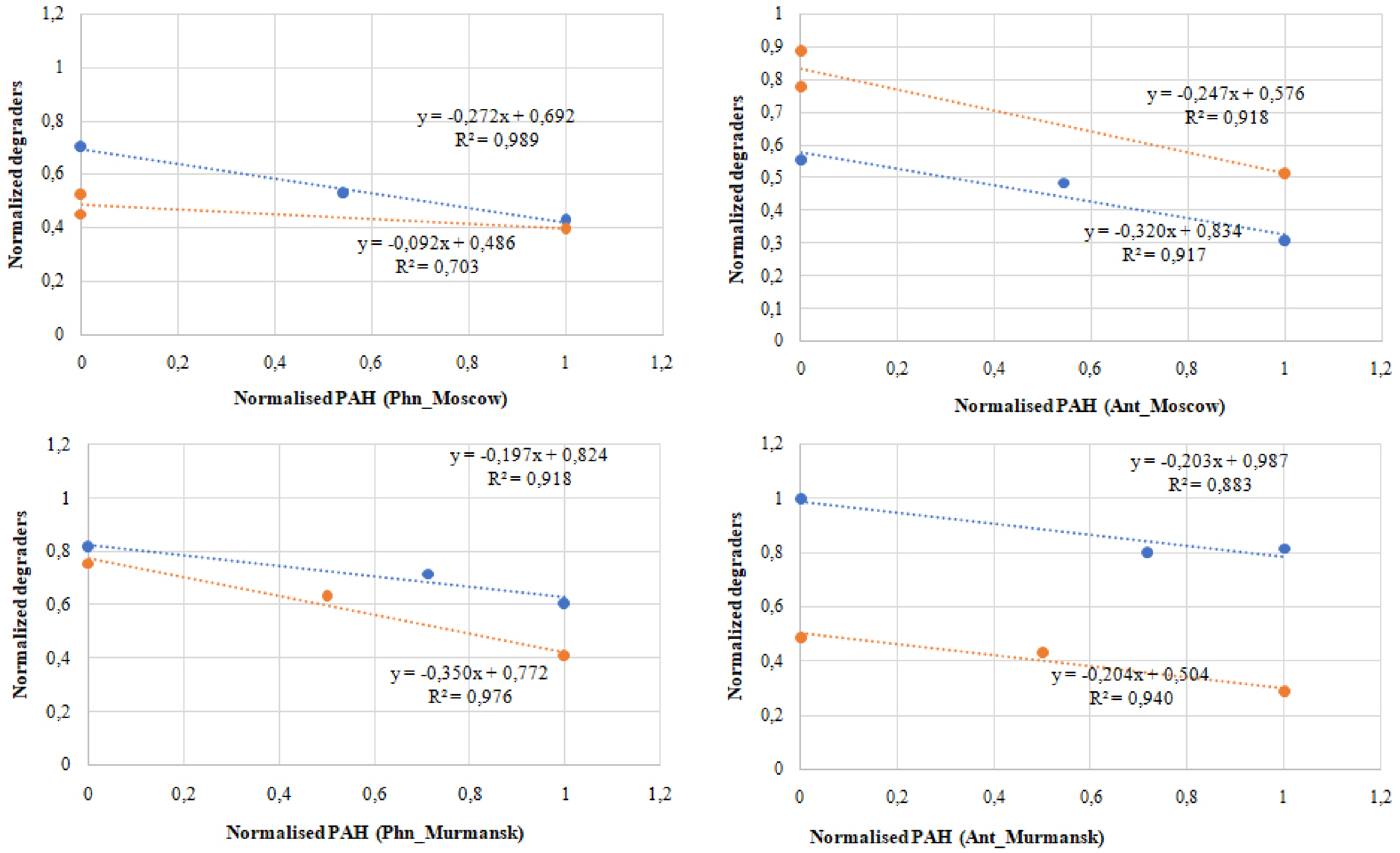

Figure S7. Linear regression analysis between normalized PAH (Ant-anthracene and Phn-phenanthrene) and normalized amount of *Micrococcus* degrader in the three urban sites of Moscow and Murmansk of leaf (blue) and road (orange) dust. Statistical significance of R-squared coefficient was reported ($p < 0.05$).
