# Supplementary figures and images for: Polycyclic Aromatic Hydrocarbon-Degrading Bacteria in Three Different Functional Zones of the Cities of Moscow and Murmansk
Source: Microorganisms. 2022 Oct 6;10(10):1979. doi: 10.3390/microorganisms10101979 (PMC9610461; doi:10.3390/microorganisms10101979)

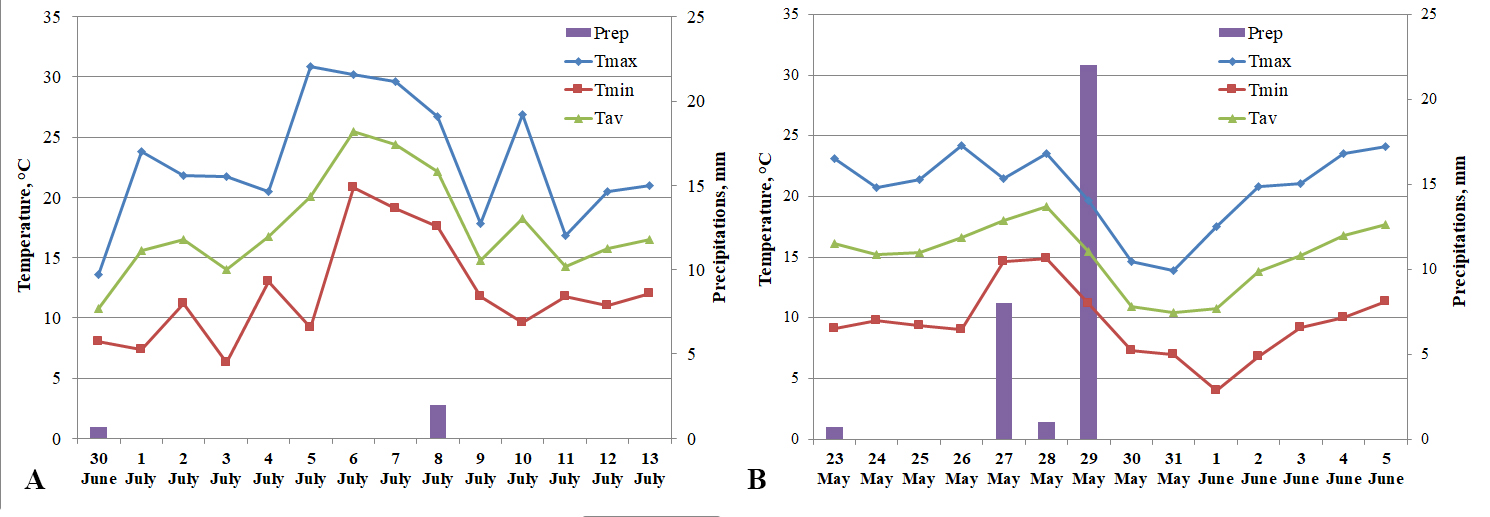

Supplement: Supplementary file 1 [file microorganisms-10-01979-s001.zip › Figure S1.jpg]

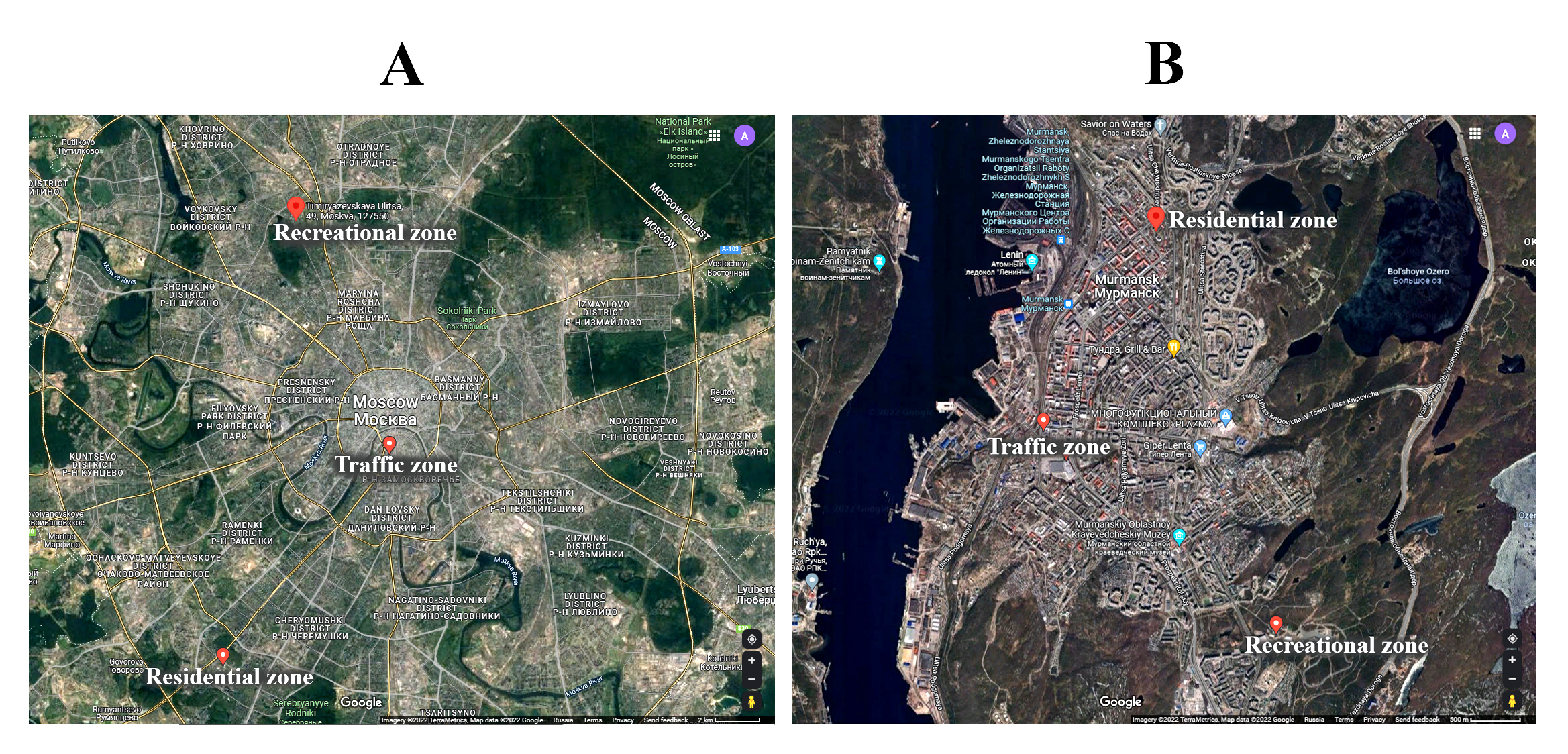

Supplement: Supplementary file 1 [file microorganisms-10-01979-s001.zip › Figure S2.jpg]

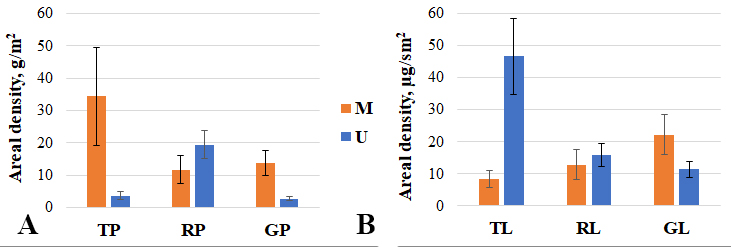

Supplement: Supplementary file 1 [file microorganisms-10-01979-s001.zip › Figure S3.jpg]

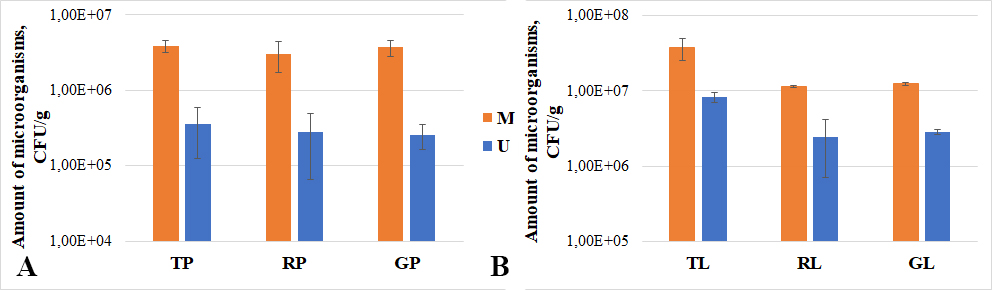

Supplement: Supplementary file 1 [file microorganisms-10-01979-s001.zip › Figure S4.jpg]

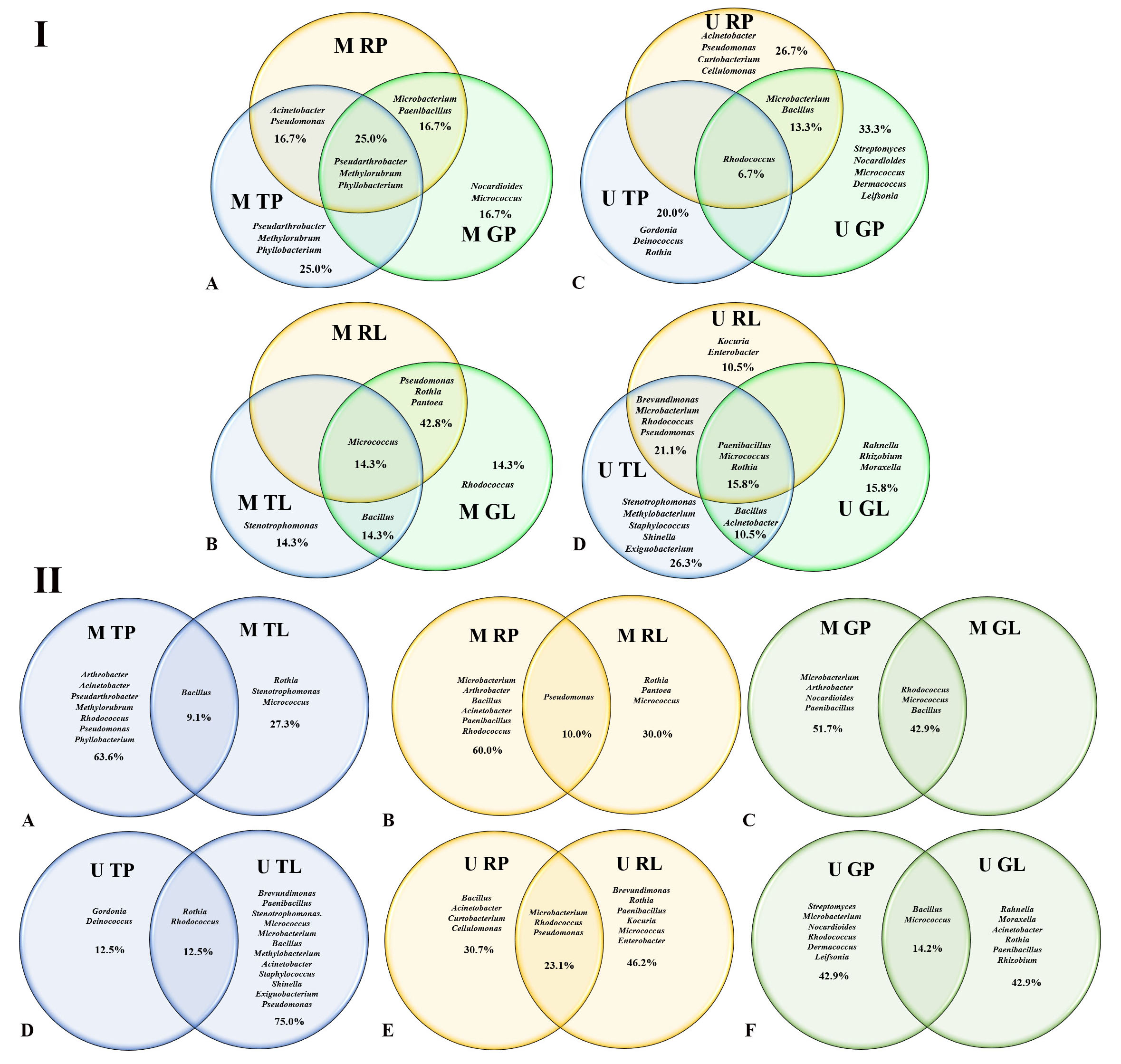

Supplement: Supplementary file 1 [file microorganisms-10-01979-s001.zip › Figure S5.jpg]

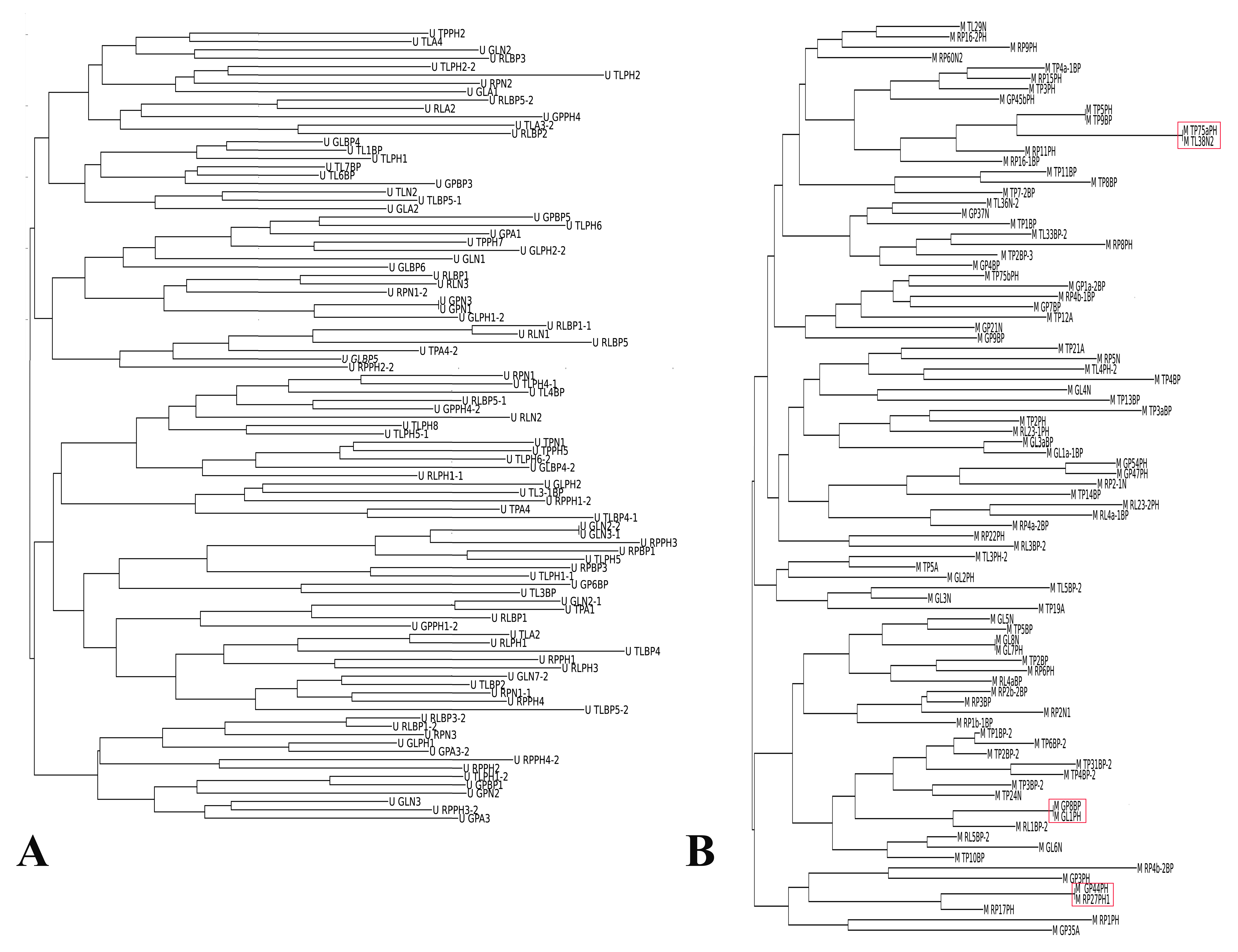

Supplement: Supplementary file 1 [file microorganisms-10-01979-s001.zip › Figure S6.jpg]

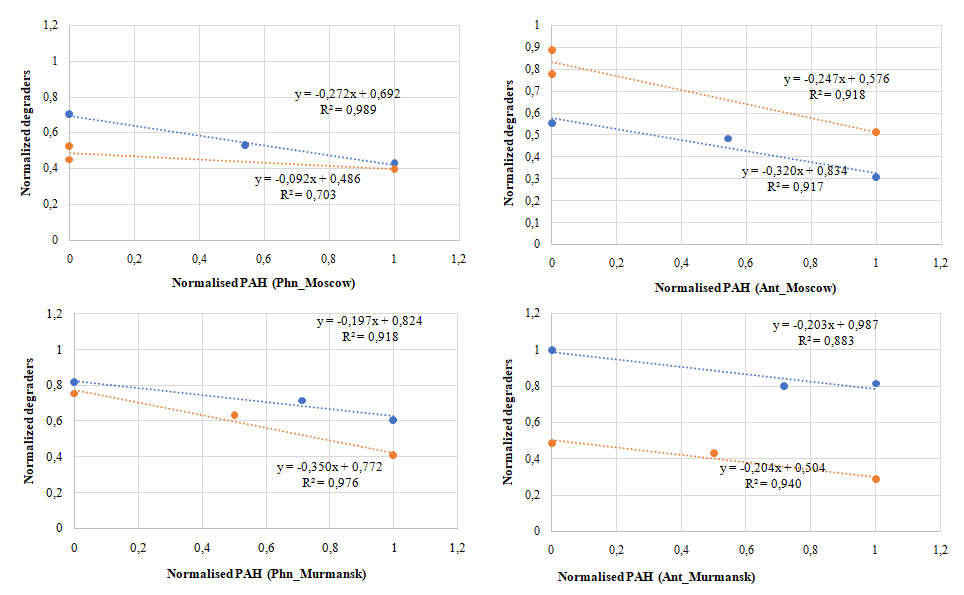

Supplement: Supplementary file 1 [file microorganisms-10-01979-s001.zip › Figure_S7.jpg]
